# Prediction models for mortality in patients with sepsis: a systematic review and meta-analysis

**DOI:** 10.3389/fmed.2026.1730156

**Published:** 2026-06-10

**Authors:** Siyuan Lei, Huanrong Ruan, Jun Wang, Guixiang Zhao, Hulei Zhao, Jianping Liu, Jiansheng Li

**Affiliations:** 1 Lung Disease Diagnosis and Treatment Center, National Medical Center, The First Affiliated Hospital of Henan University of Chinese Medicine, Zhengzhou, China; 2 Collaborative Innovation Center for Chinese Medicine and Respiratory Diseases Co-constructed by Henan Province & Education Ministry of P.R. China/Henan Key Laboratory of Chinese Medicine for Respiratory Diseases, Henan University of Chinese Medicine, Zhengzhou, China; 3 Centre for Evidence-Based Medicine, Hospital of Chengdu University of Traditional Chinese Medicine, Chengdu, China; 4 Centre for Evidence-Based Medicine, Beijing University of Chinese Medicine, Beijing, China

**Keywords:** meta-analysis, mortality, predictive model, sepsis, systematic review

## Abstract

**Background:**

Sepsis remains a leading cause of mortality among critically ill patients worldwide. Although an increasing number of prediction models have been published in recent years, their predictive performance, methodological quality, and major predictors have not been comprehensively evaluated in a systematic and quantitative manner. This study aims to evaluate the performance of these models and to identify common predictors associated with sepsis mortality.

**Methods:**

We systematically searched PubMed, Embase, Cochrane Library, and Web of Science for studies on sepsis mortality prediction models published up to July 1, 2025. Data were extracted and appraised using the Checklist for Critical Appraisal and Data Extraction for Systematic Reviews of Prediction Modeling Studies (CHARMS), and risk of bias was assessed with the Prediction Model Risk of Bias Assessment Tool for Artificial Intelligence (PROBAST+AI). Meta-analyses were performed to pool area under the curve of the receiver operating characteristic (AUC) metric of externally validated models and the odds ratio (OR) of common predictors. The study was registered in PROSPERO (CRD42024604119).

**Results:**

A total of 84 eligible studies were included, reporting 235 prediction models for sepsis mortality and involving approximately 2.7 million patient records reported across studies, with 461,387 deaths. Only 11(13.10%) studies encompassed model development, internal validation, and external validation. The included studies comprised 78(92.86%) retrospective cohort studies, 57(67.86%) studies developed in intensive care unit (ICU) settings, with MIMIC databases being among the most commonly used data sources. The most prevalent mortality endpoints were in-hospital (*n* = 38, 45.24%), 28-day (*n* = 23, 27.38%), and 30-day mortality (*n* = 16, 19.05%). The included studies employed various modeling approaches, such as logistic regression (LR), random forest (RF), eXtreme Gradient Boosting (XGBoost), and K-nearest neighbors (KNN). Of the included studies, 46(54.76%) evaluated the calibration, 26(30.95%) conducted decision curve analysis, and 25(29.76%) applied SHAP for interpretability, while most did not provide visual model presentation (e.g., nomograms). The reported AUCs ranged from 0.590–0.976 for model development and 0.530–0.992 for internal validation. The pooled AUC of externally validated models, based on one representative model per study, was 0.794 (95% CI: 0.755–0.834), indicating moderate discriminative performance. Overall, the most commonly used predictors were age (*n* = 19, 22.62%), lactate (*n* = 13, 15.48%), albumin (*n* = 8, 9.52%), SOFA score (*n* = 8, 9.52%), and vasopressor (*n* = 7, 8.33%). Notably, 64 (76.19%) studies and 50 (65.79%) studies were judged to have a high risk of bias in model development and model evaluation phases, respectively.

**Conclusion:**

Externally validated prediction models generally demonstrate moderate discriminative performance for predicting sepsis mortality, but a substantial proportion of these studies were evaluated as having a high risk of bias. Age, lactate, albumin, SOFA score, and vasopressor use were identified as predictors of mortality. Future studies with larger cohorts, rigorous designs, and multicenter external validation are warranted to improve their generalizability and facilitate clinical implementation.

**Systematic review registration:**

The unique registration identifier is CRD42024604119, and the publicly accessible website is https://www.crd.york.ac.uk/prospero/.

## Introduction

Sepsis is a life-threatening organ dysfunction caused by a dysregulated host response to infection, characterized by high incidence, elevated mortality, and substantial economic burden, which remains the leading cause of death for critically ill patients worldwide (1, 2). In 2017, the World Health Organization (WHO) declared that improving the prevention, recognition, and management of sepsis is a global health priority (3). Globally, approximately 48.9 million cases of sepsis and 11 million sepsis-related deaths were recorded, accounting for 19.7% of all global deaths (4). A recent updated meta-analysis showed a hospital mortality rate of 26.7% among sepsis patients, while 41.9% of those treated in intensive care units (ICUs) died before discharge (5). Considering the ongoing demographic shift toward an aging population, it is anticipated that the incidence and mortality of sepsis will increase in the future (6). Therefore, early prediction of mortality risk in patients with sepsis is of great clinical significance, as it enables accurate prognostic assessment, supports clinical decision-making, and ultimately contributes to reducing mortality.

At present, various scoring systems have been widely applied for clinical prognosis assessment in sepsis, including the SOFA score (7), APACHE II score (8), and SAPS II score (9), which have been proven to effectively assist clinicians in evaluating disease severity and predicting mortality risk. However, these scoring tools were not specifically designed for sepsis (10), and their clinical application may be limited by complex calculation methods, insufficient individual specificity, and considerable subjectivity (11, 12). Rapid advancements in machine learning have provided powerful tools for early disease prevention and diagnosis (13), assessment of treatment efficacy (14), and improvement of patient prognosis (15). Machine learning-based models have shown the potential to improve predictive performance and may offer advantages over conventional clinical scoring systems in certain settings (16). A systematic review of existing sepsis mortality prediction models could identify gaps for future studies and provide clinicians with a robust basis for recognizing patients at high risk of death.

In recent years, several systematic reviews and meta-analyses on sepsis prediction models have been published (17–19). Fleuren et al. (17) extracted data from 28 studies involving 130 models, and demonstrated that machine learning-based models could accurately predict the occurrence of sepsis. However, their work primarily focused on early diagnosis of sepsis rather than mortality risk. Another study by Zhang et al. (18) comprehensively reviewed 104 prediction models from 50 studies and suggested that machine learning outperformed conventional clinical scoring tools in predicting sepsis mortality. Nonetheless, it mainly emphasized methodological aspects of machine learning, included many models developed for predicting mortality risk for sepsis-related comorbidities, and did not conduct meta-analysis of predictors, all of which may compromise the accuracy of the conclusions. Nikravangolsefid et al. (19) indicated that machine learning models have shown potential improvement in predicting sepsis mortality, while its scope was restricted to models developed in ICU settings and lacked a quantitative pooled analysis. It is noteworthy that several sepsis mortality risk models with rigorous designed studies and favorable predictive performance have been published during 2024–2025. Therefore, we conducted this systematic review and meta-analysis to comprehensively evaluate the performance and methodological quality of multivariable prediction models for sepsis mortality and to identify the most important predictors determining model performance.

## Materials and methods

This systematic review and meta-analysis was conducted in accordance with the Preferred Reporting Items for Systematic Reviews and Meta-Analysis statement (PRISMA 2020) (20). The review protocol was designed according to the Checklist for critical Appraisal and data extraction for systematic Reviews of prediction Modeling Studies (CHARMS) (21) and the Guidelines for Systematic Reviews and Meta-Analyses of Prediction Model Performance (22), and was prospectively registered in the International Prospective Register of Systematic Reviews (PROSPERO) (CRD42024604119).

### Search strategy

We systematically searched PubMed, Embase, the Cochrane Library, and Web of Science from database inception to July 1, 2025, to identify all studies developing and/or validating a mortality prediction model in sepsis patients. Search terms included the Medical Subject Headings (MeSH) terms and free-text keywords, and the search algorithm was as follows: (“sepsis” OR “septic shock” OR “severe sepsis” OR “systemic inflammatory response syndrome” OR “SIRS”) AND (“prediction model” OR “prognostic model” OR “prediction tool” OR “risk prediction” OR “risk score” OR “risk calculation” OR “risk assessment” OR “machine learning” OR “deep learning”) AND (“mortality” OR “death” OR “prognosis”). In addition, we further hand screened the references lists of all included articles and relevant systematic reviews to identify potential additional eligible studies. Details of the literature search strategy are presented in Supplementary Table 1.

### Inclusion and exclusion criteria

The inclusion criteria were as follows: (1) the primary purpose of the study was to develop or validate a model for predicting the risk of mortality in sepsis patients. (2) the included studies were retrospective or prospective cohorts. (3) the target subjects of the models should be adult patients explicitly diagnosed with sepsis (including sepsis, severe sepsis or septic shock). (4) The patient settings were not limited to in-hospital, emergency department, or ICUs. (5) Eligible outcomes encompassed mortality of sepsis patients occurring at any time point, such as in-hospital mortality, 28-day mortality, 30-day mortality.

The exclusion criteria were as follows: (1) studies primarily aimed at developing or validating diagnostic models for sepsis, methodological studies, and studies purely screening for risk factors. (2) studies developing models using only one predictor. (3) To increase population comparability, we excluded studies focusing on comorbidities of sepsis with other diseases such as pneumonia-associated sepsis and sepsis with heart failure. (4) studies that are non-English, reviews, letters, conference abstracts, study protocols, case studies, duplicate publications, incomplete data or no relevant outcome.

### Study selection

According to the predefined inclusion and exclusion criteria, two reviewers independently screened the deduplicated records. Initially, irrelevant studies were excluded based on titles and abstracts. Subsequently, the full texts of all potentially eligible articles were assessed to determine final inclusion. Any issues or discrepancies encountered during the screening process were resolved through discussion with a third reviewer until consensus was reached.

### Data extraction

In accordance with the recommendations of the CHARMS checklist, two reviewers independently performed data extraction using a predesigned standardized form. For all included studies, we extracted the following information: first author, year and journal of publication, country, study design, model type, study population, data source, study period, predicted outcomes, definition of sepsis, clinical setting, modeling approach, total sample size and number of events, number and types of predictors in the final model, measures of predictive performance (AUC, sensitivity, specificity, etc), internal validation methods, variable selection strategies, handling of missing data, calibration methods, assessment of clinical usefulness, model presentation format, and model name. Notably, if an article reported multiple models, we extracted data separately for each model.

### Risk of bias assessment

Two reviewers independently applied the Prediction Model Risk of Bias Assessment Tool for Artificial Intelligence (PROBAST+AI) (23) to appraise the risk of bias and applicability of the included studies. This comprehensive tool is an extension of the original PROBAST (24) tool and is specifically designed to evaluate prediction models developed using regression or artificial intelligence methods. Following the PROBAST+AI framework, the assessment was conducted separately for two distinct phases: model development and model evaluation. For each phase, the risk of bias was evaluated across four domains: (1) participants and data sources, (2) predictors, (3) outcome, and (4) analysis. Each signaling question within a domain was answered as “yes,” “probably yes,” “probably no,” “no,” “no information” or “not applicable.” If any question within a domain was signaled as “no” or “probably no,” the domain was considered to be at high risk of bias. Conversely, a domain was considered to be at low risk of bias if all signaling questions were answered as “yes” or “probably yes.” The overall risk of bias was graded as low risk when all four domains were judged to be at low risk. In addition, applicability was assessed independently of the risk of bias and comprised three domains: participants and data sources, predictors, and outcomes.

### Statistical analysis

A qualitative synthesis was conducted to summarize the basic characteristics of the included studies and the prediction models. We performed a random-effects meta-analysis of the AUC values for all included external validation studies. Studies reporting a single external validation dataset were included directly. When a study reported multiple models in external validation, only one representative model was selected to ensure statistical independence, in accordance with the CHARMS guideline recommendations. To minimize selection bias, we preferentially selected the model identified by the authors as the primary or recommended model. In cases where no primary model was specified, the best-performing model was chosen to represent the study, consistent with approaches adopted in previous meta-analyses (25). If measures of uncertainty were not reported, 95% confidence intervals(95%CI) for AUC values were approximated using established formulas based on the number of events and the total sample size (26, 27). The Chi-square test and *I*^2^ were used to quantify heterogeneity between studies. Sensitivity analyses were conducted to assess the robustness of the overall results and to explore potential sources of heterogeneity. We implemented funnel plots and Egger’s regression test to detect publication bias. Furthermore, a meta-analysis of single proportions was performed to estimate pooled sepsis mortality rates across studies, and adjusted odds ratios (ORs) with 95% CI were synthesized for predictive factors. To address the issue of overlapping cohorts, duplicate populations were de-duplicated during quantitative synthesis. For studies originating from the same cohort, only a single representative study was included in the meta-analysis, preferentially selecting the study with the largest sample size. The synthesis of mortality rates and predictor effects was pre-specified in the PROSPERO protocol, whereas the pooled analysis of externally validated AUC values was further specified during the review process within the pre-defined objective of evaluating predictive performance. All statistical analyses were performed using the Stata software (version 18.0).

## Results

### Literature screening

**Figure 1:**
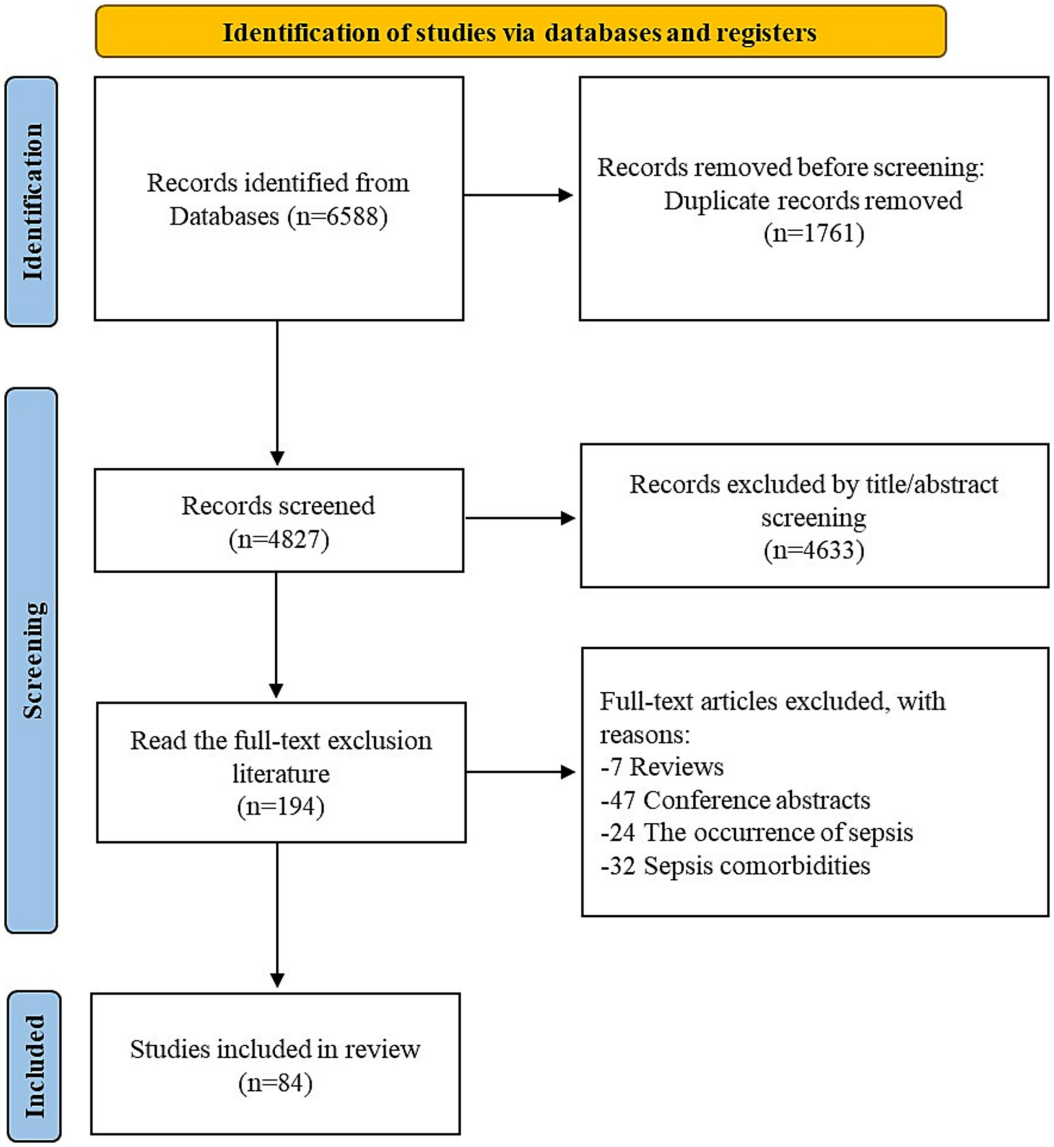
PRISMA flow diagram of the study selection process.

Of the 6,588 screened articles, 1,761 duplicate articles were removed, 4,633 articles were excluded by reading the titles and abstract, and 110 articles were excluded after full text review (Seven reviews, 47 conference abstracts, 24 predictions of sepsis occurrence, and 32 sepsis comorbidities). Ultimately, 84 studies (28–111) met the inclusion and exclusion criteria. The PRISMA flow diagram of the study selection process is shown in Figure 1.

### Study characteristics

Our review included 84 studies (28–111) reporting 235 mortality prediction models in sepsis patients, involving more than 2.7 million patient records reported across studies, with 461,387 deaths. Of the included studies, eight studies were limited to model development, 59 studies conducted both model development and internal validation, six studies conducted model development and external validation, and 11 studies comprehensively covered model development, internal validation, and external validation. These studies were published from 2009 to 2025, comprised 78 retrospective cohort studies, four prospective cohort studies, and two prospective-retrospective cohort studies, which including 51 multi-center and 33 single-center investigations. These models were mainly developed in China (*n* = 56; 66.67%), the United States (*n* = 7; 8.33%), South Korea (*n* = 3; 3.57%), Colombia (*n* = 3; 3.57%), and Spain (*n* = 3; 3.57%). Medical Information Mart for Intensive Care II (MIMIC-II), MIMIC-III and MIMIC-IV emerged as the predominant data sources employed for developing models in 36 of the 84 included studies, which underscores the potential for overlapping cohorts across studies. Among the included studies, 65 studies used the Sepsis-3 criteria, 10 studies used the ICD-9/ICD-10 to define sepsis, and nine studies used other definitions of sepsis. The included studies were developed in a variety of clinical settings, 57 studies were conducted in ICU, 18 in emergency departments (ED), four in the inpatient wards, three in both ED and ICU, while two studies did not specify the specific setting. Regarding primary outcomes, 38 studies evaluated in-hospital mortality, 23 studies focused on 28-day mortality, and 16 studies used 30-day mortality as the primary outcome. The basic characteristics of the included studies are shown in Supplementary Table 2.

Various modeling methods were applied, including Logistic Regression(LR), Cox Regression, Random Forest (RF), eXtreme Gradient Boosting (XGBoost), K-nearest neighbor (KNN), Naive Bayes (NB), Support Vector Machine (SVM), Long short-term memory(LSTM), Gradient boosting Machine(GBM), Tree-based methods (Decision Tree(DT) and Classification And Regression Tree(CART)), Neural network-based models (including Artificial Neural Networks (ANN), Deep Neural Network (DNN), and Multi-layer perceptron neural network (MLP)), and Blending model. Twenty-five models used cross-validation, whereas the remaining models had random split validation (*n* = 34; 40.48%), bootstrapping (*n* = 2; 2.38%), or a combination of methods (*n* = 9; 10.71%). Calibration was assessed in 46 studies, of which 22 used calibration curves or plots, 12 applied the Hosmer-Lemeshow test, two employed the Brier score, and 10 adopted multiple methods. The majority of models performed variable selection before including predictors into the final model. Logistic regression was the most frequently applied method (*n* = 32;38.10%), followed by least absolute shrinkage and selection operator (Lasso) regression (*n* = 12;14.29%) and univariable analysis (*n* = 7,8.33%), while a smaller portion identified predictors based on prior knowledge or machine learning techniques. A substantial number of models (*n* = 60;71.43%) did not provide any form of model presentation, whereas 20 reported presenting the model using a nomogram, and only four provided the regression equation. Eighteen applied multiple imputation, 11 used mean or median imputation, and five adopted KNN imputation. In addition, only 26 studies performed decision curve analysis, and 25 utilized the SHapley Additive exPlanations (SHAP) algorithm to interpret model outputs. Details of the model assessment information for the included studies are shown in Supplementary Table 3.

### Model performance

For the evaluation of model performance, beyond the AUC, several studies employed other measures such as accuracy, sensitivity, specificity, positive predictive value (PPV), negative predictive value (NPV), F1 score. A total of 59 studies provided AUC for model development, ranging from 0.590 to 0.976, and 65 studies reported internal validation AUCs ranging from 0.530 to 0.992. Regarding other performance metrics of model development, 19 studies reported accuracy (0.515–0.968), 21 reported sensitivity (0.509–0.942), 20 reported specificity (0.307–0.942), 11 reported PPV (0.480–0.965), 10 reported NPV (0.112–0.963), and nine reported the F1 score (0.507–0.989). For internal validation, 29 studies documented accuracy (0.337–0.980), 23 documented sensitivity (0.330–0.970), 24 documented specificity (0.344–0.970), 13 documented PPV (0.216–0.896), 13 documented NPV (0.210–0.990), and 19 documented the F1 score (0.271–0.960). In addition, 40 of the 84 studies reported comparisons of prediction models with conventional severity scoring systems such as SOFA, APS-III, SAPS-II, and APACHE II. The AUC values of conventional scoring systems ranged from 0.542 to 0.832. Detailed information on the model development and the internal validation of the model performance are presented in Supplementary Table 4.

### Mortality predictors

The development of predictive models in these studies incorporated a wide range of variables, ranging from three to 188, including laboratory findings, vital signs, demographic characteristics, comorbidities, and other relevant factors. At the study level, predictive factors used more than five times included age (*n* = 19; 22.62%), lactate (*n* = 13; 15.48%), albumin (*n* = 8; 9.52%), SOFA score (*n* = 8; 9.52%), vasopressor use (*n* = 7; 8.33%), comorbidities with metastatic cancer (*n* = 6; 7.14%), GCS score (*n* = 6; 7.14%), TBIL (*n* = 5; 5.95%), PLT (*n* = 5; 5.95%), and shock (*n* = 5; 5.95%). The predictors included in the models are summarized in Supplementary Table 5.

### External validation studies

External validation was performed for only 18 studies (28, 33, 40, 41, 53, 60, 63, 69, 70, 73, 74, 79, 82, 96, 102, 106, 108, 109) (21.43%), including 14 studies using completely independent datasets, 3 using institutional/geographical split validation, and 1 using temporal split validation. Among the 18 external validation studies, sample sizes ranged from 75 to 662,739, which encompassed a total of 816,683 participant records reported across studies, with 97,716 deaths. Eight studies reported their predominant data sources from publicly available critical care databases such as MIMIC-III, MIMIC-IV, and eICU, while seven studies sourced data from hospital-based cohorts. The AUC for mortality prediction across these studies ranged from 0.618 to 0.973. Details of the external validation studies of prediction models are presented in Supplementary Table 6.

### Risk of bias assessment

Using the PROBAST+AI tool, we assessed the risk of bias and applicability of all included studies, with detailed results presented in Supplementary Table 7. Regarding model development, 64 studies were judged to be at high risk of bias, while only one study was identified as having high concern regarding applicability. For model evaluation, 50 studies were assessed as having a high risk of bias, and one study demonstrated high concern for applicability. Overall, a substantial proportion of the included studies were rated as having a high risk of bias in both the model development and evaluation phases.

### Meta-analysis

#### Meta-analysis of external validation prediction models

**Figure 2:**
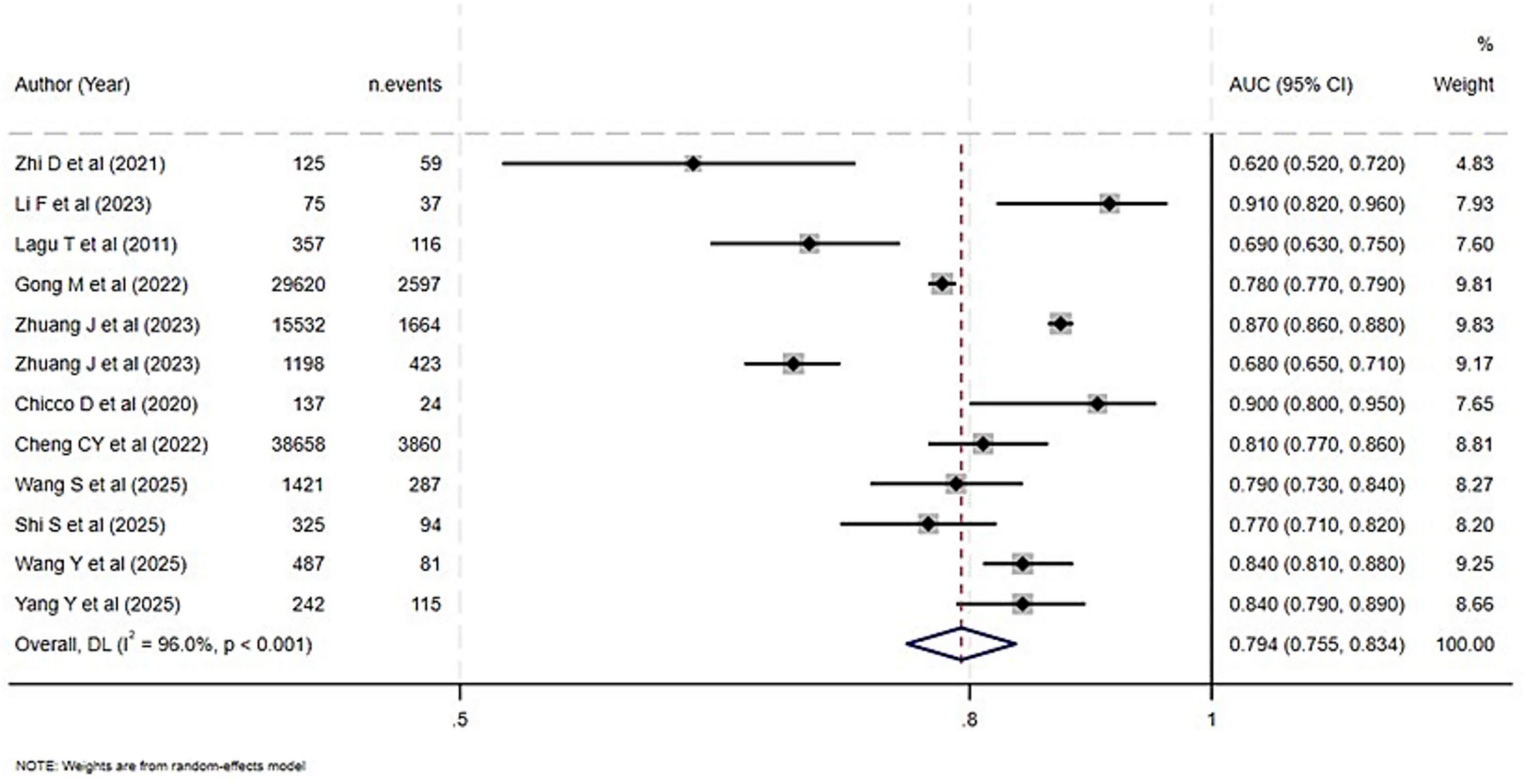
Forest plot of mortality prediction models in external validation studies.

**Figure 3:**
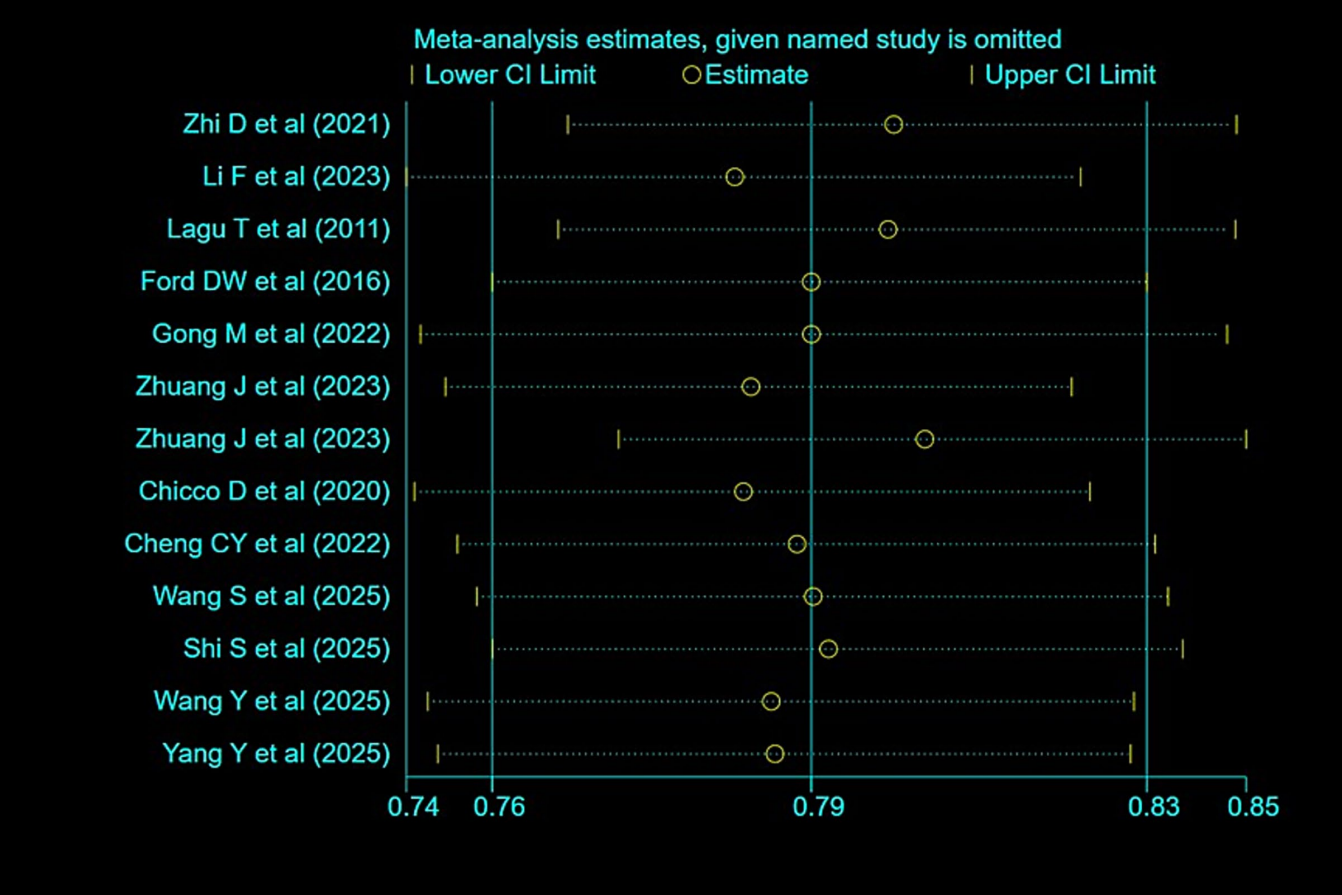
Sensitivity analysis of mortality prediction models in external validation studies.

**Figure 4:**
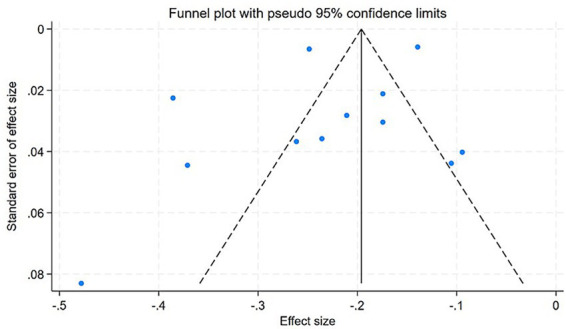
Funnel plot of mortality prediction models in external validation studies.

After accounting for potential overlapping cohorts, we conducted a meta-analysis of the AUC values and corresponding 95% CI from 18 externally validated studies in our review. Notably, Zhuang et al. (69) performed external validation using three distinct population datasets, so three separate AUC estimates were extracted and treated as independent validation datasets for analysis. The pooled AUC was calculated using a random effects model, resulting in a value of 0.794 (95% CI: 0.755–0.834; Figure 2). Given the substantial heterogeneity (*I*^2^ = 96.0%, *p* < 0.001), we conducted a sensitivity analysis by omitting each study to explore potential sources of heterogeneity, and the results indicated that the overall findings were relatively robust (Figure 3; Supplementary Table 8). Furthermore, to address the potential heterogeneity arising from different sepsis definitions, a sensitivity analysis restricted to studies adhering to the Sepsis-3 criteria was performed. The pooled AUC value was 0.803 (95% CI: 0.763–0.845), suggesting that variation in sepsis definitions had a limited impact on the main outcome (Supplementary Figure 1). Publication bias was assessed using a funnel plot, which showed an approximately symmetrical distribution of the scatter points (Figure 4). Furthermore, Egger’s regression test (*p* = 0.375) suggested no significant evidence of publication bias among the included studies. After excluding overlapping cohorts and selecting one representative externally validated model per study to reduce non-independence and double counting, the pooled AUC should be interpreted as a broad summary of model discrimination across heterogeneous external validation settings, rather than as a precise common performance estimate applicable to all sepsis populations.

#### Meta-analysis of mortality rates in sepsis patients

Of the included studies, 23 studies reported 28-day mortality, 16 studies reported 30-day mortality, two studies reported 90-day mortality, three studies reported 1-year mortality, and 38 studies reported in-hospital mortality in sepsis patients. After de-duplication of overlapping cohorts, the estimated pooled mortality of sepsis was 25.5% (95% CI 23.9–27.0%, *I*^2^ = 99.9%, *p*<0.001). Separate random-effects meta-analyses were conducted according to different follow-up periods. The pooled mortality was 30.2% (95% CI: 26.7–33.7%, *I*^2^ = 98.5%, *p*<0.001) for 28-day mortality, 21.1% (95% CI: 15.7–26.5%, *I*^2^ = 98.7%, *p*<0.001) for 30-day mortality, 43.6%(19.4–67.7%, *I*^2^ = 94.2%, *p*<0.001) for 90-day mortality, 49.7% (95% CI: 37.2–62.2%, *I*^2^ = 99.0%, *p*<0.001) for 1-year mortality, and 20.2% (95% CI: 17.8–25.5%, *I*^2^ = 99.9%, *p*<0.001) for in-hospital mortality. Detailed results are presented in Supplementary Table 9 and Supplementary Figure 2.

#### Meta-analysis of the predictive factors involved in the predictive model

We analyzed the predictive value of seven frequently reported predictors (appearing in more than five studies), including age, lactate, albumin, SOFA score, vasopressor use, metastatic cancer, and GCS score. After de-duplication of overlapping cohorts, the meta-analysis demonstrated that age (OR = 1.07, 95% CI: 1.04–1.09, *I*^2^ = 95.8%, *p*<0.001), lactate (mmol/L) (OR = 1.29, 95% CI: 1.19–1.41, *I*^2^ = 93.9%, *p*<0.001), albumin (g/dL) (OR = 0.61, 95% CI: 0.49–0.77, *I*^2^ = 93.7%, *p*<0.001), SOFA score (OR = 1.14, 95% CI: 1.07–1.21, *I*^2^ = 80.0%, *p*<0.001), vasopressor use (OR = 2.25, 95% CI: 1.89–2.67, *I*^2^ = 81.8%, *p*<0.001), comorbidities with metastatic cancer (OR = 1.95, 95% CI: 1.53–2.47, *I*^2^ = 91.4%, *p*<0.001), and GCS score (OR = 0.91, 95% CI: 0.88–0.94, *I*^2^ = 76.7%, *p*<0.001) were significantly associated with mortality risk in patients with sepsis. Detailed meta-analysis results are presented in Table 1 and Supplementary Figure 3.

**Table 1:** The meta-analysis results for these predictors in the included studies.

Predictors	Model	Pooled estimate	Heterogeneity	
		(95% CI)	I^2^	*P*
Age	Random	1.07(1.04–1.09)	95.8%	<0.001
Lactate	Random	1.29(1.19–1.41)	93.9%	<0.001
Albumin	Random	0.61(0.49–0.77)	93.7%	<0.001
SOFA score	Random	1.14 (1.07–1.21)	80.0%	<0.001
Vasopressor	Random	2.25 (1.89–2.67)	81.8%	<0.001
Comorbidities with metastatic cancer	Random	1.95 (1.53–2.47)	91.4%	<0.001
GCS score	Random	0.91 (0.88–0.94)	76.7%	<0.001

CI, Confidence interval; OR, Odds ratio. To facilitate interpretation, pooled ORs for continuous variables represent the risk associated with a per-unit increase (e.g., per 1-year increase for age, per 1 mmol/L increase for lactate, per 1 g/dL increase for albumin, and per 1-point increase for SOFA and GCS scores).

## Discussion

This systematic review and meta-analysis included 84 original studies reporting 235 models for predicting sepsis mortality, encompassing a total of more than 2.7 million patient records. After rigorously accounting for overlapping cohorts, the pooled AUC of the externally validated models included in meta-analysis was 0.794 (95% CI, 0.755–0.834), indicating moderate discriminatory performance. Although the pooled AUC was calculated after excluding overlapping cohorts and selecting one representative externally validated model per study, substantial heterogeneity remained (*I*^2^ = 96.0%). Therefore, this estimate should not be interpreted as a precise or universally generalizable measure of model performance. Rather, it provides a broad indication that externally validated models generally showed moderate discrimination, while the magnitude of performance may vary considerably across populations, data sources, sepsis definitions, outcome time points, and validation designs. The pooled sepsis mortality rate was 25.5% (95% CI, 23.9–27.0%), and the most frequently identified predictors included age, lactate, albumin, SOFA score, etc. However, given the substantial heterogeneity between studies, the high risk of bias in most included studies, and the limited number of models subjected to rigorous external validation, these findings should be interpreted with caution.

### Principal findings

The pooled in-hospital, 28-day, 30-day, 90-day, and 1-year mortality rates of sepsis in included studies were 20.2, 30.2, 21.1, 43.6, and 49.7% respectively, which indicates that sepsis mortality remains relatively high and generally increases with longer follow-up, which is broadly consistent with previous studies (112, 113). Prospective data collection represents the optimal approach for developing robust prediction models (114), while only 4(4.8%) studies adopted such a design in our review, which may restrict the generalizability and applicability of the results to other patient populations and clinical settings. In our review, 36 of 84 studies (42.86%) used MIMIC-II, MIMIC-III, or MIMIC-IV as the primary data source (115, 116). This concentration of evidence in a small number of publicly available ICU databases may amplify dataset-specific patient profiles, coding structures, clinical practice patterns, variable availability, and model development strategies. Even after de-duplication of overlapping cohorts, such overrepresentation may still bias the evidence base and limit broader generalizability to non-ICU settings, non-US healthcare systems, and prospectively collected real-world cohorts. There was inconsistency in the definitions of sepsis across the included studies, while 65 studies adopted the Sepsis-3.0 criteria, 19 studies relied on ICD codes or other guideline-based definitions (117). These alternative approaches may not reliably identify sepsis patients and could contribute to heterogeneity in the pooled results. Such inconsistency may also alter the apparent importance of predictors by changing the underlying case-mix and severity spectrum of the included populations. Future model development should strictly adhere to the latest Sepsis-3 criteria to ensure consistency and clinical applicability.

Before prediction models can be implemented in clinical practice, they must be externally validated in independent populations with different clinical characteristics, and their predictive performance should be compared to identify those with the best discrimination and calibration (118). External validation is essential for evaluating the generalizability and applicability of models across various clinical settings and patient populations. However, only six studies conducted both model development and external validation, and only eleven studies completed the full process of model development, internal validation, and external validation in our review. This limitation reduces the reliability and external generalizability of the pooled performance estimates and restricts their immediate clinical applicability. Therefore, the pooled performance should be considered a summary of the currently available validation evidence rather than proof of readiness for routine clinical implementation. This imbalance indicates that the primary challenge in the field is no longer the development of new models, but rather the lack of rigorous, multicenter external validation of existing ones. Continued development of new models based on similar datasets and study designs may contribute little to clinical translation unless issues of generalizability and calibration are adequately addressed. Future research should prioritize external validation, updating, and comparative evaluation of high-performing models across diverse clinical settings, which is essential to bridge the current gap between data science and clinical critical care medicine. Selecting appropriate variables is a critical step for improving the predictive accuracy of models. Most studies employed logistic regression for variable selection, but some relied solely on univariable analysis, which may overlook the combined effects of multiple predictors (119). During model development process, variable selection strategies should be optimized by incorporating prior knowledge and using multivariable approaches to reduce the number of predictors and minimize the risk of overfitting (24). In addition, approximately half of the studies either did not report any methods of handling missing data or directly deleted incomplete cases. Failure to appropriately impute missing data may impair the model performance, particularly when the missingness is associated with other observed characteristics (120).

Calibration reflects the extent to which a model accurately estimates absolute risk, assessing whether the predicted values correspond to the observed outcomes. Poor calibration may lead to underestimation or overestimation of the target outcome. Among the included studies, only 46 reported calibration using calibration plots, the Hosmer-Lemeshow test, or the Brier score, while a substantial proportion did not provide calibration (121). Moreover, only a quarter of the included studies conducted decision analysis. In general, discrimination and classification statistics that were reported in studies cannot inform about the clinical value of a prediction model, so decision analysis is required to evaluate the cost-effectiveness of implementing the sepsis mortality risk prediction models in practice (122). Therefore, the limited and inconsistent reporting of calibration and clinical utility prevents a comprehensive assessment of model usefulness beyond discrimination, and acceptable AUC values alone should not be taken as sufficient evidence for clinical implementation. Models with acceptable discrimination should instead be considered potentially promising rather than clinically ready unless adequate calibration and net clinical benefit are demonstrated in independent external validation cohorts. In addition, SHAP is a commonly used tool for assessing feature importance in machine learning models. By applying a game-theoretic framework, it quantifies the contribution of each feature to the model’s final output, thereby addressing the opacity of machine learning algorithms and enhancing the global interpretability of the model (123). Our systematic review revealed that only a limited number of prognostic models performed SHAP for model interpretation. The visual presentation of prediction models is a key factor in acceptance among healthcare professionals, with common formats including nomograms, risk charts, decision trees, and sum scores. However, most models did not provide any form of representation, which may hinder the understanding and application of model outputs in clinical decision-making (124).

The predictive factors included in this systematic review encompassed demographic characteristics, vital signs, laboratory results, comorbidities, and other relevant factors. Different prediction models emphasized distinct aspects and incorporated a wide range of indicators. Our meta-analysis indicated that age is a major risk factor for mortality in sepsis, with the risk of death increasing progressively with advancing age, which is consistent with previous findings. This association may be attributed to impaired immune responses in older individuals, such as inadequate leukocyte antigen processing and altered expression of inflammatory cytokines (125). Lactate is another prognostic indicator of mortality in patients with sepsis. Current guidelines also regard lactate as an essential diagnostic criterion for septic shock, and it plays a pivotal role in guiding sepsis management, underscoring the need for prompt lactate assessment at admission and heightened attention to those patients with elevated levels (126). Albumin is the most abundant protein in plasma, and our review demonstrated that low albumin levels are closely associated with increased mortality in sepsis patients, which aligns with the findings of most previous research (1). 27.85 g/L has been proposed as a target level for albumin infusion within the first seven days to improve the prognosis of septic patients (127). This meta-analysis also confirmed that the SOFA score remains a robust and widely accepted standard for predicting sepsis mortality, owing to its comprehensive multisystem assessment and consistency with previous evidence (128). The use of vasopressors is one of the diagnostic criteria for septic shock and usually occurs when initial aggressive goal-directed fluid resuscitation fails to maintain adequate circulatory stability, thereby necessitating vasopressor administration to ensure sufficient tissue perfusion pressure. This intervention may be associated with poorer clinical outcomes (109). It is noteworthy that comorbidities with metastatic cancer, GCS score, TBIL, PLT, and shock are also important factors influencing mortality in sepsis. These variables should be given due consideration in the subsequent development of predictive models.

Unfortunately, the risk of bias was substantial: 64 of 84 studies (76.19%) were judged to have a high risk of bias in model development, and 50 studies were judged to have a high risk of bias in model evaluation. These methodological limitations lower confidence in the reported model performance and may lead to optimism in discrimination estimates, unstable predictor effects, and overestimation of clinical applicability. Overall, the primary sources of bias arose from three domains: analysis, predictors, and the participants and data sources. These issues included inappropriate internal and external validation methods, inadequate model performance evaluation, insufficient sample sizes, failure to appropriately handle missing data, inconsistent definitions and measurement methods of predictors, reliance on univariable analyses for predictor selection, and unclear inclusion and exclusion criteria for study participants. According to the PROBAST+AI guidelines, an adequate sample size should be ensured during model development to support robust model construction and reduce the risk of overfitting. The criterion of at least 10 outcome events per candidate variable can serve as a minimum reference threshold (129). It is important to note that candidate variables refer to all predictors considered at any stage of the modeling process, rather than only those included in the final model (130). Many studies have shown a high risk of bias due to an excessive number of candidate predictors combined with insufficient sample sizes. Furthermore, variations in clinical settings, along with differences in the number, type, and the timing of variable collection between studies, may influence the performance of predictive models. Accordingly, the pooled performance estimates and identified predictors should be interpreted cautiously, as the high risk of bias may reduce their robustness and clinical applicability.

### Strengths and limitations

This study has several strengths. Firstly, we conducted a comprehensive literature search of multiple databases in accordance with PRISMA guidelines. Study selection and data extraction were independently performed by two reviewers, and detailed quality assessment and risk of bias evaluation were conducted following the TRIPOD and PROBAST+AI checklists. These methodological approaches enhanced the ability to accurately catalog the entire information on evaluation of the mortality risk prediction models for sepsis patient. Secondly, our review supplements and extends the evidence provided by previous systematic reviews (18, 19). Compared with earlier reviews (18, 19), our study provides a detailed description of model characteristics, performs a meta-analysis of AUC values for externally validated models, quantifies the prognostic impact of included predictors, and incorporates the most recent research published in the current year. These strengths offer valuable insights for the design of future studies on prediction models for sepsis mortality and provide a robust foundation for the selection of reliable prognostic factors in model development.

Admittedly, our review also has several potential limitations. First, the included studies were restricted to those published in English, which may have introduced selection bias. Second, although primary models recommended by the original authors were preferentially selected, reliance on the best-performing models when no primary model was specified may still introduce selection bias and overestimate overall discrimination. However, given the limited number of externally validated studies and methodological heterogeneity, more advanced approaches such as hierarchical modeling were not feasible. Third, the reporting quality of calibration was generally poor, making a quantitative synthesis of model calibration infeasible. This was mainly because calibration was assessed using heterogeneous methods, including calibration plots, the Hosmer-Lemeshow test, and the Brier score, while key quantitative measures required for synthesis were rarely reported in a consistent and extractable form. In addition, substantial heterogeneity was observed not only in the pooled AUC estimates of externally validated models, but also in the pooled mortality rates and predictor effects. This likely reflects marked clinical and methodological diversity across studies. Due to the limited number of studies available for each specific pooled analysis and the substantial clinical and methodological heterogeneity across included studies, we were unable to perform reliable meta-regression or subgroup analyses to further explore the sources of heterogeneity. Therefore, these pooled estimates should be interpreted with appropriate caution. Taken together, the very high heterogeneity, dataset concentration, limited external validation, and high risk of bias reduce the certainty of the pooled estimates and limit the extent to which a single summary value can be applied to individual clinical settings. Therefore, the pooled AUC, mortality rates, and predictor effects should be regarded as descriptive summary estimates rather than definitive performance or effect estimates applicable to all sepsis populations.

## Conclusion

This systematic review and meta-analysis synthesized evidence from 84 studies on prediction models for sepsis mortality and suggested that externally validated models generally showed moderate discriminative performance. However, the certainty and clinical applicability of this finding remain limited by substantial heterogeneity, dataset concentration, limited rigorous external validation, and high risk of bias across studies. Future studies should adopt multicenter prospective designs with adequate sample sizes, appropriately handle continuous variables and missing data, conduct rigorous internal and external validation, comprehensively evaluate calibration, clinical utility, and cost-effectiveness, and present models in visual formats to facilitate clinical interpretation and ultimately improve the prognosis of patients with sepsis.

## Data Availability

The original contributions presented in the study are included in the article/Supplementary material, further inquiries can be directed to the corresponding author/s.

## Glossary

AUC: Area under the Receiver Operating Characteristics
PPV: Positive predictive value
NPV: Negative predictive value
LR: logistic regression model
RF: Random Forest
XGBoost: eXtreme Gradient Boosting
KNN: K-nearest neighbor
NB: Naive Bayes
SVM: Support vector machine
CART: Classification and Regression Tree
MARS: Multivariate Adaptive Regression Splines
ANN: Artificial neural network
GBM: Gradient boosting Machine
TCN: Temporal convolutional network
LSTM: Long short-term memory
Cox: Cox proportional regression hazards model
GBDT: Gradient boosting decision tree
DNN: Deep Neural Network
GCN: Graph Convolutional Network
DT: Decision tree
MLP: Multi-layer perceptron neural network
GCS: glasgow coma scale
DCADecision curve analysis
LASSO: Least Absolute Shrinkage and Selection Operator
SHAP: the SHapley Additive exPlanations
