# Supplementary material for: Prediction models for mortality in patients with sepsis: a systematic review and meta-analysis
Source: Front Med (Lausanne). 2026 Jun 10;13:1730156. doi: 10.3389/fmed.2026.1730156 (PMC13290529; doi:10.3389/fmed.2026.1730156)
Supplement: Supplementary file 1 [file Table_1.DOC]

**Supplementary Table 1**

**Details of the literature search strategy**

(1) PubMed

| **Search** | **Query** | **Items found** |
| --- | --- | --- |
| #1 | (("Sepsis"[Mesh]) OR "Shock, Septic"[Mesh]) | 148016 |
| #2 | (((((((Sepsis*[Title/Abstract]) OR (Septicemia*[Title/Abstract])) OR (Septic shock*[Title/Abstract])) OR (Severe sepsis*[Title/Abstract])) OR (Systemic Inflammatory Response Syndrome[Title/Abstract])) OR (SIRS[Title/Abstract])) OR (septic[Title/Abstract])) OR (septicaemic shock[Title/Abstract]) | 193318 |
| #3 | #1OR#2 | 267728 |
| #4 | ((((((((prediction model[Title/Abstract]) OR (prognostic model[Title/Abstract])) OR (prediction tool[Title/Abstract])) OR (risk prediction[Title/Abstract])) OR (risk score[Title/Abstract])) OR (risk calculation[Title/Abstract])) OR (risk assessment[Title/Abstract])) OR (machine learning[Title/Abstract])) OR (deep learning[Title/Abstract]) | 357184 |
| #5 | "Mortality"[Mesh] | 433283 |
| #6 | (death[Title/Abstract]) OR (prognosis[Title/Abstract]) | 1487005 |
| #7 | #5 OR #6 | 1786807 |
| #8 | #3 AND #4 AND #7 | 808 |

(2) Embase

| **Search** | **Query** | **Items found** |
| --- | --- | --- |
| #1 | 'sepsis'/exp | 371541 |
| #2 | 'septic shock'/exp | 78168 |
| #3 | sepsis*:ab,ti OR septicemia*:ab,ti OR 'septic shock*':ab,ti OR 'severe sepsis*':ab,ti OR 'systemic inflammatory response syndrome':ab,ti OR sirs:ab,ti OR septic:ab,ti OR 'septicaemic shock':ab,ti | 286847 |
| #4 | #1 OR #2 OR #3 | 457617 |
| #5 | 'prediction model':ab,ti OR 'prognostic model':ab,ti OR 'prediction tool':ab,ti OR 'risk prediction':ab,ti OR 'risk score':ab,ti OR 'risk calculation':ab,ti OR 'risk assessment':ab,ti OR 'machine learning':ab,ti OR 'deep learning':ab,ti | 411572 |
| #6 | 'mortality'/exp | 1507532 |
| #7 | death:ab,ti OR prognosis:ab,ti | 2096411 |
| #8 | #6 OR #7 | 3220276 |
| #9 | #4 AND #5 AND #8 | 2159 |

(3) Cochrane Library

| **Search** | **Query** | **Items found** |
| --- | --- | --- |
| #1 | MeSH descriptor: [Sepsis] explode all trees | 6615 |
| #2 | MeSH descriptor: [Shock, Septic] explode all trees | 1439 |
| #3 | (Sepsis*):ti,ab,kw OR (Septicemia*):ti,ab,kw OR (Septic shock*):ti,ab,kw OR (Severe sepsis*):ti,ab,kw OR (Systemic Inflammatory Response Syndrome):ti,ab,kw OR (SIRS):ti,ab,kw OR (septic):ti,ab,kw OR (septicaemic shock):ti,ab,kw | 20483 |
| #4 | #1 OR #2 OR #3 | 22517 |
| #5 | (prediction model):ti,ab,kw OR (prognostic model):ti,ab,kw OR (prediction tool):ti,ab,kw OR (risk prediction):ti,ab,kw OR (risk score):ti,ab,kw OR (risk calculation):ti,ab,kw OR (risk assessment):ti,ab,kw OR (machine learning):ti,ab,kw OR (deep learning):ti,ab,kw | 220873 |
| #6 | MeSH descriptor: [Mortality] explode all trees | 18997 |
| #7 | (death):ti,ab,kw OR (prognosis):ti,ab,kw | 137779 |
| #8 | #6 OR #7 | 146618 |
| #9 | #4 AND #5 AND #8 | 1565 |

(4) [Web of Science](https://apps.webofknowledge.com/home.do?SID=6BQQjiiMCVa9MgFvRpC) core collection

| **Search** | **Query** | **Items found** |
| --- | --- | --- |
| #1 | Topic: (Sepsis* OR Septicemia* OR "Septic shock*" OR "Severe sepsis*" OR "Systemic Inflammatory Response Syndrome" OR SIRS OR septic OR "septicaemic shock") | 256209 |
| #2 | Topic: ("prediction model" OR "prognostic model" OR "prediction tool" OR "risk prediction" OR "risk score" OR "risk calculation" OR "risk assessment" OR "machine learning" OR "deep learning") | 1003422 |
| #3 | Topic: (mortality OR death OR prognosis) | 2986677 |
| #4 | #1 AND #2 AND #3 | 2056 |
