# Supplementary material for: Prediction models for mortality in patients with sepsis: a systematic review and meta-analysis
Source: Front Med (Lausanne). 2026 Jun 10;13:1730156. doi: 10.3389/fmed.2026.1730156 (PMC13290529; doi:10.3389/fmed.2026.1730156)
Supplement: Supplementary file 2 [file Table_2.DOC]

**Table 1** **The basic characteristics of the included studies**

| **Author** | **Year** | **Journal** | **Study design** | **Model type** | **Country** | **Study period** | **Dataset source** | **Sepsis definition** | **Sepsis type** | **Setting** | **Primary outcome** | **Outcome cases/sample size** | **Age**  **(Mean**  **/Median)** | **Gender**  **(Male/Female)** |
| --- | --- | --- | --- | --- | --- | --- | --- | --- | --- | --- | --- | --- | --- | --- |
| Zhi D et al^[8]^ | 2021 | American Journal of Infection Control | Retrospective,Multi-center | D,E | China | 2001-2012 | MIMIC-Ⅲ | Sepsis 3.0 | Sepsis | ICU | 30-day mortality | 862/1,964 | - | 1,126/838 |
| Zhang G et al^[9]^ | 2024 | European Journal of Medical Research | Retrospective, Multi-center | D,I | China | 2008-2019,2014-2015,2003-2016 | MIMIC-IV, eICU-CRD, AmsterdamUMCdb | Sepsis 3.0 | Sepsis | ICU | In-hospital mortality | 555/3,535 | 66 | 1,977/1,558 |
| Yu Z et al^[10]^ | 2024 | BMC Medical Informatics and Decision Making | Retrospective, Multi-center | D,I | China | 2001-2012 | MIMIC-III | Sepsis 3.0 | Sepsis | ICU | 30-day mortality | 1,787/9,118 | 65.13 | 5,161/3,957 |
| Li Y et al^[11]^ | 2024 | Scientific Reports | Retrospective,Multi-center | D,I | China | 2001-2012 | MIMIC-III | Sepsis 3.0 | Sepsis | ICU | In-hospital mortality | 1,926/9,432 | 63.16 | - |
| Xu Z et al^[12]^ | 2024 | PeerJ | Retrospective,Single-center | D,I | China | 2020.12-2021.09 | the Second Affiliated Hospital, School of Medicine, Zhejiang University | Sepsis 3.0 | Septic shock | ICU | 28-day mortality | 78/304 | 59.70 | 207/97 |
| Li F et al^[13]^ | 2024 | Aging (Albany NY) | Retrospective,Single-center | D | China | 2021.6-2023.8 | First Affiliated Hospital, Hengyang Medical School | Sepsis 3.0 | Sepsis | Inpatient wards | 28-day mortality | 114/456 | - | 285/171 |
| García de Guadiana-Romualdo L et al^[14]^ | 2024 | Medicine Intensiva | Retrospective,Single-center | D | Spain | 2015.1-2016.11 | Santa Lucía University Hospital (Cartagena, Spain) | Sepsis 3.0 | Sepsis, septic shock | ICU | 28-day mortality | 26/173 | 67 | 109/64 |
| Li F et al^[15]^ | 2023 | Medical Science Monitor | Retrospective,Single-center | D,E | China | 2018.1.1-2022.12.31 | the Second Affiliated Hospital of Nanchang University | Sepsis 3.0 | Sepsis | ICU | 28-day mortality | 139/415 | 56 | 248/167 |
| Wang J et al^[16]^ | 2023 | BMC Infectious Diseases | Retrospective,Multi-center | D,I | China | 2001-2012 | MIMIC-III | Sepsis 3.0 | Sepsis | ICU | 1-year mortality | 1,113/1,983 | 67.79 | 1,164/819 |
| Taylor RA et al^[17]^ | 2016 | Acad Emerg Med | Retrospective,Multi-center | D,I | USA | 2013.10-2014.10 | Four Eds, Yale University, Yale-New Haven Hospital | ICD-9 | Sepsis | ED | In-hospital mortality | 260/5,278 | - | 2,427/2,851 |
| Seo MH et al^[18]^ | 2016 | Yonsei Med J. | Prospective,Single-center | D,I | Korea | 2012.1.1-2014.12.31 | Yonsei University College of Medicine, Severance Hospital, Seoul, Kore | 2001 SCCM/ESICM/ACCP/ATS/SIS International Sepsis Definitions Conference | Severe Sepsis, Septic Shock | ED | 28-day mortality | 108/561 | - | 283/278 |
| Zhao C et al^[19]^ | 2019 | Int Immunopharmacol | Retrospective,Multi-center | D,I | China | 2001-2012 | MIMIC-Ⅲ | Sepsis 3.0 | Sepsis | ED | 28-day mortality | 1,182/5,663 | - | 2,963/2,700 |
| Zhang K et al^[20]^ | 2021 | Front Med (Lausanne) | Retrospective,Multi-center | D,E | China | 2001-2012 | MIMIC-Ⅲ | Sepsis 3.0 | Sepsis | ICU | 30-day mortality | 1,949/11,101 | - | - |
| Zeng Z et al^[21]^ | 2021 | BioData Mining | Retrospective,Multi-center | D,E | China | 2001-2012,2014-2015 | MIMIC-III and eICU-CRD | Sepsis 3.0 | Sepsis | ICU | In-hospital mortality | 4,422/24,653 | - | 13,581/11,072 |
| Zeng Q et al^[22]^ | 2021 | Biomed Res Int | Retrospective,Single-center | D,I | China | 2018.5-2020.10 | the 908th People’s Liberation Army Hospital (Nanchang, China) | Sepsis 3.0 | Sepsis | ICU | 90-Day mortality | 73/231 | 70 | 143/88 |
| Wernly B et al^[23]^ | 2020 | Int J Med Inform | Retrospective,Multi-center | D,I | Austria | 2001-2012,2014-2015 | MIMIC-III and eICU | Angus criteria | Sepsis, septic shock | ICU | 96 h mortality | 1,350/13,634 | - | 7,305/6,279 |
| Wang H et al^[24]^ | 2022 | BMC Med Inform Decis Mak | Retrospective,Multi-center | D,I | USA | 2001-2012 | MIMIC-III | Sepsis 3.0 | Sepsis, septic shock | ICU | In-hospital mortality | 836/5,783 | - | 2,562/3,221 |
| van Doorn WPTM et al^[25]^ | 2021 | PLoS One | Retrospective,Single-center | D,I | Netherlands | 2015.1.1-2016.12.31 | Maastricht University Medical Centre | Sepsis 3.0 | Sepsis, septic shock | ED | 31-day mortality | 174/1,344 | - | 719/625 |
| Su L et al^[26]^ | 2021 | Front Med (Lausanne) | Retrospective,Single-center | D,I | China | 2016-2018 | ICU of Peking Union Medical College Hospital | Sepsis 3.0 | Sepsis, septic shock | ICU | In-hospital mortality | 415/2,224 | 58.96 | 1,292/932 |
| Rodríguez A et al^[27]^ | 2021 | Am J Emerg Med | Retrospective,Multi-center | D,I | Colombia | 2014.6-2016.2 | Hospital San Vicente Fundación, Hospital Pablo Tobón Uribe and IPS Universitaria León XIII | Sepsis 1.0 | Sepsis and septic shock | ED and ICU | In-hospital mortality | 289/2,510 | 62 | 1,251/1,259 |
| Perng JW et al^[28]^ | 2019 | J Clin Med | Retrospective,Multi-center | D,I | Taiwan | 2007.1-2013.12 | Chang Gung Research Database | Sepsis 1.0 | Sepsis and septic shock | ED | 72 h and 28-day mortality | 72h:1,991/42,220;28day:5,939/42,220 | - | 22,208/20,012 |
| Park JY et al^[29]^ | 2022 | J Med Internet Res. | Retrospective,Multi-center | D,I | USA | 2010-2014 | the US National Inpatient Sample database | Angus criteria | Sepsis | - | In-hospital mortality | 196,841/923,759 | 67.94 | 468,295/455,464 |
| Liu N et al^[30]^ | 2021 | PLoS One | Retrospective,Single-center | D,I | Singapore | 2014.9-2017.4 | Singapore General Hospital | Sepsis 3.0 | Sepsis | ED | 30-day mortality | 66/342 | - | 174/168 |
| Liu H et al^[31]^ | 2021 | J Int Med Res | Retrospective,Multi-center | D,I | China | 2001-2012 | MIMIC III | ICD-9 | Sepsis, severe sepsis, and septic shock | ICU | 30-day,60-day and 90-day mortality | -/5,240 | - | 2,907/2,333 |
| Li K et al^[32]^ | 2021 | Medicine (Baltimore) | Retrospective,Multi-center | D,I | China | 2001-2012 | MIMIC-III | ICD-9 | Sepsis, severe sepsis, and septic shock | ICU | In-hospital mortality | 1,352/3,937 | - | 2,212/1,725 |
| Lagu T et al^[33]^ | 2011 | Crit Care Med | Retrospective,Multi-center | D,E | USA | 2004.6.1-2006.6.30 | Perspective database (Premier Healthcare Informatics, Charlotte, NC) | ICD-9 | Sepsis | ICU | In-hospital mortality | 33,308/167,288 | - | 80,875/86,413 |
| Kong G et al^[34]^ | 2020 | BMC Med Inform Decis Mak | Retrospective,Multi-center | D,I | China | 2001–2012 | MIMIC III | Sepsis 3.0 | Sepsis | ICU | In-hospital mortality | 2,949/16,688 | 65.61 | 9,087/7,601 |
| Karlsson A et al^[35]^ | 2021 | BMC Emerg Med | Retrospective,Single-center | D,I | Sweden | 2013.1.1-2013.12.31 | Södersjukhuset in Sweden | ICD-10 | Sepsis, Severe sepsis | ED | 7-day and 30-day mortality | day:63/445;30day:98/445 | 73 | 234/211 |
| Hu C et al^[36]^ | 2022 | Infect Dis Ther | Retrospective,Multi-center | D,I | China | 2008-2019 | MIMIC-IV | Sepsis 3.0 | Sepsis | ICU | In-hospital mortality | 1,107/8,817 | 66.8 | 5,456/3,361 |
| Hou N et al^[37]^ | 2020 | J Transl Med | Retrospective,Multi-center | D,I | China | 2001–2012 | MIMIC III | Sepsis 3.0 | Sepsis, severe sepsis, septic shock | ICU | 30-day mortality | 889/4,559 | - | 2,537/2,022 |
| Hargovan S et al^[38]^ | 2021 | Aust Crit Care | Retrospective,Single-center | D,I | Australia | 2014.1.1-2018.6.1 | Cairns Hospital ICU electronic medical record database | Sepsis 3.0 | Sepsis, septic shock | ICU | In-hospital mortality | 59/500 | 58 | 272/228 |
| García-Gallo JE et al^[39]^ | 2020 | Med Intensiva (Engl Ed) | Retrospective,Multi-center | D,I | Colombia | 2001–2012 | MIMIC III | ICD-9 | Sepsis, severe sepsis, septic shock | ICU | 1-year mortality | 2,450/5,650 | 67.54 | 3,084/2,566 |
| Ford DW et al^[40]^ | 2016 | Crit Care Med | Retrospective,Multi-center | D,I,E | USA | 2012 | Acute care, non-federal hospitals in NY, MD, FL, MI, and WA | ICD-9 | Severe sepsis, septic shock | ED | In-hospital mortality | 109,934/771,179 | - | 379,221/391,958 |
| Phillips GS et al^[41]^ | 2018 | Crit Care Med | Retrospective,Multi-center | D,I | USA | 2015.1.1-2015.12.31 | 179 hospitals across New York State | Sepsis 2.0 | Severe sepsis, septic shock | - | In-hospital mortality | -/43,204 | - | - |
| Ribas Ripoll VJ et al^[42]^ | 2014 | Artif Intell Med | Retrospective,Multi-center | D,I | Spain | 2001-2008 | MIMIC II | 2001 SCCM/ESICM/ACCP/ATS/SIS International Sepsis Definitions Conference | Sepsis, severe sepsis, septic shock | ICU | In-hospital mortality | 84/400 | 65.2 | 225/175 |
| Gong M et al^[43]^ | 2022 | Med Biol Eng Comput | Retrospective,Multi-center | D,I,E | China | 2008–2019,2014-2015 | MIMIC-IV and EICU | Sepsis 3.0 | Sepsis | ICU | In-hospital mortality | 5,113/46,140 | 65.15 | 27,195/18,945 |
| García-Gallo J E et al^[44]^ | 2019 | Int. J. Pharma Med. Biol. Sci. | Retrospective,Multi-center | D,I | Colombia | 2001–2012 | MIMIC III | Sepsis 3.0 | Sepsis,Severe sepsis,and Septic Shock | ICU | 1-year mortality | 2,446/5,650 | 67.54 | - |
| Wang W et al^[45]^ | 2021 | Revista Română de Medicină de Laborator | Retrospective,Single-center | D | China | 2017.8-2021.1 | Department of Emergency, Tianjin | Sepsis 3.0 | Septic shock | ED | 28-day mortality | 31/118 | 65.74 | 67/51 |
| Ding X et al^[46]^ | 2022 | Int Immunopharmacol | Retrospective,Single-center | D | China | 2019.3-2019.9 | First Affiliated Hospital of Zhengzhou University | Sepsis 3.0 | Sepsis | ICU | 28-day, in-hospital mortality, and 90-day mortality | In hospital:53/96; 28day:49/96 90day:54/96 | - | 57/39 |
| Wang L et al^[47]^ | 2022 | Signa Vitae | Prospective, Single-center | D | China | 2018.6-2019.6 | Renji Hospital, Shanghai Jiao Tong University School of Medicine | Sepsis 3.0 | Sepsis, septic shock | ED | 28-day mortality | 41/175 | 66 | 110/65 |
| Cheng YW et al^[48]^ | 2024 | J Clin Monit Comput | Retrospective,Multi-center | D,I | China,Taiwan | 2008–2019 | MIMIC-IV | Sepsis 3.0 | Sepsis | ICU | 30-day mortality | 2,799/24,377 | - | 11,106/8,037 |
| Zhuang J et al^[49]^ | 2023 | BMC Med Inform Decis Mak | Retrospective,Multi-center | D,I,E | China, Hong Kong | 2001–2012, 2008–2019, 2014–2015, 2019–2020 | MIMIC-III, MIMIC-IV, eICU, Zigong | Sepsis 3.0 | Sepsis | ICU | In-hospital mortality | 7,326/56,088 | - | 31,635/24,453 |
| Zheng F et al^[50]^ | 2023 | Biomedical Signal Processing and Control | Retrospective,Multi-center | D,I,E | China | 2008–2019, 2014–2015 | MIMIC-IV,eICU | Sepsis 3.0 | Septic shock | ICU | 28-day mortality | 2,273/11,947 | - | - |
| Pan X et al^[51]^ | 2023 | BMC Infect Dis | Retrospective,Multi-center | D,I | China | 2008-2019 | MIMIC-IV | Sepsis 3.0 | Sepsis | ICU | In-hospital mortality | 3,659/23,889 | 65.06 | 13,800/10,089 |
| Li S et al^[52]^ | 2023 | J Clin Med | Retrospective,Multi-center | D,I | China | 2008-2019 | MIMIC-IV | Sepsis 3.0 | Sepsis | ICU | In-hospital mortality | 3,759/24,272 | 66.1 | 14,060/10,212 |
| Bao C et al^[53]^ | 2022 | Medicina Intensiva | Retrospective,Multi-center | D,E | China | 2008-2019,2014-2015 | MIMIC-IV, eICU | Sepsis 3.0 | Sepsis | ICU | In-hospital mortality | 3,771/21,680 | 67 | 11,960/9,750 |
| Wang ZY et al^[54]^ | 2022 | Chin Med Sci J | Retrospective,Multi-center | D,I,E | China | 2008-2019 | MIMIC-IV | Sepsis 3.0 | Sepsis | ICU | 30-day mortality | 2,915/12,664 | 66.45 | 5,249/7,415 |
| Su Y et al^[55]^ | 2022 | Eur J Med Res | Retrospective,Multi-center | D,I | China | 2001-2012 | MIMIC-III | ICD-9 | Sepsis | ICU | 30-day mortality | 856/2,874 | 67 | 1,602/1,272 |
| Ke X et al^[56]^ | 2022 | Comput Math Methods Med | Retrospective,Multi-center, | D,I | China | 2008–2019 | MIMIC-IV | Sepsis 3.0 | Elderly sepsis | ICU | In-hospital mortality | 2,845/18,522 | - | 10,355/8,167 |
| Zhang Z et al^[57]^ | 2017 | Oncotarget | Retrospective,Multi-center | D,I | China | 2001-2012 | MIMIC-III | ICD-9 | Severe sepsis | ICU | In-hospital mortality | 1,054/3,206 | 73.29 | - |
| Wang M et al^[58]^ | 2023 | Infect Drug Resist | Retrospective,Single-center | D,I | China | 2013.1-2023.1 | Dataset from affiliated Dongyang Hospital of Wenzhou Medical University (China) | Sepsis 3.0 | Septic shock | ED | In-hospital mortality | 384/1,253 | 73 | 749/504 |
| Chicco D et al^[59]^ | 2020 | Sci Rep | Retrospective,Multi-center | D,I,E | Canada | 2011–2012, 2007–2015 | Norwegian Patient Registry & Statistics Norway; South Korean critically ill patient dataset | Sepsis3.0 | Sepsis, Septic shock | inpatient wards | In-hospital mortality | 11,735/110,341 | - | 58,063/52,278 |
| Adrie C et al^[60]^ | 2009 | Crit Care | Prospective,Multi-center | D,I | France | 1996.11-2007.4 | Multicentre database (OUTCOMEREA®) | 2001 SCCM/ESICM/ACCP/ATS/SIS International Sepsis Definitions Conference | Severe sepsis, septic shock | ICU | 14-day mortality | 630/2,268 | - | - |
| Chen SH et al^[61]^ | 2021 | Tzu Chi Med J | Retrospective,Single-center | D | Taiwan,China | 2009.1-2011.12 | Surgical ICUs, National Taiwan University Hospital | Sepsis 3.0 | Sepsis | ICU | 28-day mortality | 211/739 | 64.8 | 504/235 |
| Cheng CY et al^[62]^ | 2022 | Front Med (Lausanne) | Retrospective,Multi-center | D,I,E | Taiwan,China | 2006-2017 | Chang Gung Medical Center database (5 EDs, Taiwan) | Sepsis 3.0 | Sepsis | ED | In-hospital mortality | 19,434/193,646 | - | - |
| Gao J et al^[63]^ | 2024 | BMC Med Inform Decis Mak | Retrospective,Multi-center | D,I | USA | 2008-2019 | MIMIC-IV | Sepsis 3.0 | Sepsis | ICU | In-hospital mortality | 2,120/7,304 | 65.3 | 4,229/3,075 |
| Greco, M et al^[64]^ | 2023 | Algorithms | Retrospective,Single-center | D,I | Italy | - | Humanitas Research Hospital ED HER | Sepsis 3.0 | Sepsis, septic shock | ED | In-hospital mortality | 65/425 | 77 | 249/176 |
| He B et al^[65]^ | 2024 | Front Artif Intell | Retrospective,Multi-center | D,I | China | 2001-2012 | MIMIC-III | Sepsis 3.0 | Sepsis, septic shock | ICU | 28-day mortality | 903/5,834 | 66 | 3,492/2,342 |
| Hong C et al^[66]^ | 2024 | Ther Clin Risk Manag | Retrospective,Single-center | D,I | China | 2016.12-2019.7 | Shenzhen People’s Hospital | Sepsis 3.0 | Sepsis, septic shock | ICU | In-hospital mortality | 382/1,733 | 61.1 | 1,013/720 |
| Jeon E et al^[67]^ | 2023 | Signa Vitae | Retrospective,Single-center | D,I | Korea | 2016.1-2020.2 | Registry from ED of Korea University Medical Center | Sepsis 3.0 | Sepsis, septic shock | ED | 30-day mortality | 259/810 | 75 | 473/337 |
| Jiang Z et al^[68]^ | 2023 | Comput Methods Programs Biomed | Retrospective,Multi-center | D,I | China | 2008-2019 | MIMIC-IV | Sepsis 3.0 | Sepsis | ICU | In-hospital mortality | 471/2,480 | - | - |
| Koozi H et al^[69]^ | 2023 | J Intensive Care Soc | Retrospective,Multi-center | D,I | Sweden | 2015-2018 | four mixed surgical and medical ICUs in Sweden | Sepsis 3.0 | Sepsis, septic shock | ICU | 30-day mortality | 502/1,984 | 69 | 1,153/831 |
| Li M et al^[70]^ | 2022 | J Intensive Med | Retrospective,Multi-center | D | China | 2015.9-2020.6 | Three ICUs in China (Shanghai, Jiangsu) | Sepsis 3.0 | Sepsis, septic shock | ICU | 28-day mortality | 176/545 | 68 | 341/204 |
| Li Y et al^[71]^ | 2023 | Heliyon | Retrospective +prospective,  Single-center | D,I | China | 2020.1-2020.12, 2021.1-2021.12, 2022.4-2022.5 | First Affiliated Hospital of Xinjiang Medical University | Sepsis 3.0 | Sepsis, septic shock | ED and ICU | 28-day mortality | 155/382 | 63 | 210/172 |
| Lin XM et al^[72]^ | 2024 | Heliyon | Retrospective,Single-center | D,I | China | 2018.1-2022.12 | Zhongshan Hospital, Fudan University | Sepsis 3.0 | Septic shock | ICU | 28-day mortality | 34/112 | - | 82/40 |
| Liu Y et al^[73]^ | 2022 | Cell Mol Biol (Noisy-le-grand) | Retrospective,Single-center | D,I | China | 2019.5-2022.3 | Affiliated Hospital of Xuzhou Medical University | Sepsis 3.0 | Sepsis | ICU | 28-day mortality | 40/120 | 57.3 | 87/33 |
| Lu B et al^[74]^ | 2023 | Infect Drug Resist | Retrospective,  Single-center | D,I | China | 2013.1-2022.8 | Affiliated Dongyang Hospital of Wenzhou Medical University | Sepsis 3.0 | Sepsis | Inpatient wards | In-hospital mortality | 421/1,740 | 71 | 725/1,015 |
| Park SW et al^[75]^ | 2024 | J Korean Med Sci | Prospective, Multi-center | D,I | Korea | 2019.9-2020.12 | Korean Sepsis Alliance, 19 hospitals, emergency department | Sepsis 3.0 | Sepsis, septic shock | ED | In-hospital mortality | 1,455/5,112 | - | 2,991/2,121 |
| Pérez-Tome JC et al^[76]^ | 2024 | Med Intensiva (Engl Ed) | Retrospective,  Multi-center | D,I | Spain | 2022-2023,2002-2011 | the Virgen de la Arrixaca University Hospital, Santa Lucía Hospital, and Los Arcos Hospital;MIMIC III | ICD-10 | Sepsis, severe sepsis, and septic shock | ICU | In-hospital mortality | 797/4,739 | - | - |
| Rahman MS et al^[77]^ | 2024 | BMC Med Inform Decis Mak | Retrospective,  Multi-center | D,I | Bangladesh | 2001-2012 | MIMIC-III | Sepsis 3.0 | Sepsis, septic shock | ICU | 30-day mortality | 783/4,240 | - | 2,464/1,776 |
| Wang B et al^[78]^ | 2024 | PLoS One | Retrospective,  Single-center | D,I | China | 2013.6-2021.9 | Affiliated Dongyang Hospital of Wenzhou Medical University | Sepsis 3.0 | Sepsis, septic shock | ED | 30-day mortality | 191/1,205 | 75 | 738/467 |
| Selcuk M et al^[79]^ | 2022 | Inf Med Unlocked | Retrospective,  Single-center | D,I | Turkey | 2015-2020 | Acıbadem Hospital, Istanbul-Turkey | Sepsis 3.0 | Sepsis | ICU | In-hospital mortality | 68/200 | 74.6 | - |
| Xie Y et al^[80]^ | 2023 | Eur J Clin Microbiol Infect Dis | Retrospective,  Single-center | D,I | China | 2019.9-2021.12 | Shenzhen People’s Hospital | Sepsis 3.0 | Sepsis, septic shock | ED | 28-day mortality | 53/367 | 73 | 252/115 |
| Zheng YJ et al^[81]^ | 2022 | Ann Transl Med | Retrospective,  Single-center | D,I | China | 2015.1.1-2019.12.31 | Ruijin Hospital, Shanghai Jiao Tong University School of Medicine | Sepsis 3.0 | Sepsis | inpatient wards | In-hospital mortality | 550/1,335 | 61.31 | 873/462 |
| Wang S et al^[82]^ | 2025 | NPJ Digit Med | Retrospective,  Multi-center | D,I,E | China | 2003.2-2023.11 | the electronic medical records of three hospitals in China | Sepsis 3.0 | Septic shock | ICU | 28-day mortality | 1,008/4,872 | - | 2,914/1,858 |
| Chen T et al^[83]^ | 2025 | Front Physiol | Retrospective,  Single-center | D,I | China | 2010.1-2023.8 | Longyou County People’s Hospital, Quzhou, Zhejiang, China | 2014 CN guideline +SOFA≥2 | Sepsis | ED and ICU | 30-day mortality | 173/1,050 | 74.8 | 640/410 |
| Kurtkulagi O et al^[84]^ | 2025 | Biomol Biomed | Retrospective,  Single-center | D | Türkiye | 2021.6-2023.12 | Çanakkale Onsekiz Mart University Hospital | Sepsis 3.0 | Sepsis | ICU | 28-day mortality | 109/205 | 73.6 | 109/96 |
| Li Q et al^[85]^ | 2025 | BMC Infect Dis | Retrospective,  Single-center | D,I | China | 2014.1-2023.12 | People’s Hospital of Xinjiang Uygur Autonomous Region | Sepsis 3.0 | Sepsis, septic shock | ICU | 30-day mortality | 392/1,116 | 79 | 645/471 |
| Shi S et al^[86]^ | 2025 | J Transl Med | Retrospective,  Multi-center | D,I,E | China | 2008-2019;2023.1-2023.11 | MIMIC-IV | Sepsis 3.0 | Sepsis, septic shock | ICU | In-hospital mortality | 3,419/11,808 | 59 | 7,305/4,503 |
| Shi W et al^[87]^ | 2025 | Nurs Crit Care | Retrospective,  Single-center | D,I | China | 2020.1-2022.6 | Ruijin Hospital, Shanghai Jiao Tong University | Sepsis 3.0 | Sepsis, septic shock | ICU | In-hospital mortality | 526/2,389 | 68 | 1,547/842 |
| Wang Y et al^[88]^ | 2025 | Intern Emerg Med | Retrospective,  Multi-center | D,I,E | China | 2008-2019,2021.1-2022.6 | MIMIC-IV, First Affiliated Hospital of Wenzhou Medical University | Sepsis 3.0 | Sepsis, septic shock | ICU | In-hospital mortality | 4,499/27,621 | 66.6 | 15,900/11,234 |
| Yang Y et al^[89]^ | 2025 | Sci Rep | Retrospective + prospective,  Single-center | D,I,E | China | 2018.1-2022.6,2023.1-2024.6 | ICU of tertiary hospital in Xinjiang | Sepsis 3.0 | Sepsis, septic shock | ICU | 28-day mortality | 371/822 | - | 533/289 |
| Zhang Y et al^[90]^ | 2025 | J Inflamm Res | Retrospective,  Single-center | D,I | China | 2021.8-2023.8 | Beijing Chao-Yang Hospital, Capital Medical University | Sepsis 3.0 | sepsis | ED | 28-day mortality | 120/180 | 74 | 110/70 |
| Zhu XY et al^[91]^ | 2025 | Front Physiol | Retrospective  Multi-center | D,I | China | 2008-2019 | MIMIC-IV | Sepsis 3.0 | Elderly sepsis | ICU | 28-day mortality | 1,259/4,056 | 77 | 2,192/1,864 |

Abbreviations: D, development; I, internal validation; E, external validation; ED, Emergency Department; ICU, Intensive Care Unit; SOFA, Sequential Organ Failure Assessment; -, not reported.
