# Supplementary material for: Prediction models for mortality in patients with sepsis: a systematic review and meta-analysis
Source: Front Med (Lausanne). 2026 Jun 10;13:1730156. doi: 10.3389/fmed.2026.1730156 (PMC13290529; doi:10.3389/fmed.2026.1730156)
Supplement: Supplementary file 3 [file Table_3.DOC]

**Table 2 Details of model assessment information of included studies**

| **Author** | **Sample size (D/I)** | **Performance Evaluation** | **Number of model** | **Model development method** | **Internal validation and method** | **External validation** | **Number: Variables** | **Variable selection** | **Missing data handling** | **Calibration** | **Clinical applicability** | **Model explanation** | **Model presentation** |
| --- | --- | --- | --- | --- | --- | --- | --- | --- | --- | --- | --- | --- | --- |
| Zhi D et al^[8]^ | 1,839/- | AUC, Sensitivity, Specificity, PPV, NPV, F1score | 2 | RF,LR | - | Yes | 25:(1)Demographic characteristics;(2)Clinical characteristics;(3)Rating scale for ICU. | Univariate | - | - | - | - | - |
| Zhang G et al^[9]^ | 1,132/707 | AUC, Accuracy, F1 score | 7 | XGboost,LR,RF,KNN,N B,SVM,DT | Yes,10-fold cross-validation | - | 45:(1) Laboratory blood and biochemical examination; (2)Demographics and vital signs; (3)Blood gas analysis; (4) ICU details; (5) Comorbidity and treatment modalities. | LASSO | Multiple imputation | Yes,Calibration curve | DCA | SHAP | - |
| Yu Z et al^[10]^ | 7,294/1,824 | AUC,Accuracy,F1 score | 7 | LR, LightGBM, CatBoost, RF, KNN, SVM , XGBoost | Yes,5-fold cross-validation | - | 30:(1)Demographic data; (2)Vital signs; (3)laboratory Values. | Entropy Analysis, Decision trees, clinical experts specializing in critical care | Random sampling imputation | - | - | SHAP | - |
| Li Y et al^[11]^ | 6,602/- | Accuracy | 1 | Deep Learning model | Yes,Random split validation | - | 30:(1)Laboratory indicators; (2)Vital signs. | Machine learning model, XGboost, and adopt the recommendations of clinical experts | Reference values of the indicators | - | - | - | - |
| Xu Z et al^[12]^ | 212/92 | AUC Accuracy, Sensitivity, Specificity, PPV, NPV | 1 | LR | Yes,Random split validation | - | 26:(1)Demographic data and physical condition data; (2)Laboratory data on admission; (3)Treatment method; (4)Data on length of ICU stay. | Univariate logistic regression, stepwise regression | Median imputation | - | - | - | Dynamic Nomogram |
| Li F et al^[13]^ | -/- | AUC | 1 | LR | - | - | 39 :(1)General characteristics; (2)Diagnosis at admission; (3)Laboratory examination; (4)Co-morbidities. | Multivariate logistic regression | - | - | DCA | - | Nomogram |
| García de Guadiana-Romualdo L et al^[14]^ | 173/- | AUC | 1 | LR | - | - | (1)Demographic, (2)Comorbidities, (3)Laboratory and microbiological tests and outcome data. | Multivariate logistic regression | Deletion | Yes, Hosmer-Lemeshow test | - | - | Logistic regression equation |
| Li F et al^[15]^ | 340/- | AUC,Sensitivity, Specificity | 1 | LR | - | Yes | 37 :(1)Clinical information;(2) Laboratory parameters;(3) APACHE II and SOFA score. | Multivariate logistic regression analysis | - | Yes, Hosmer-Lemeshow test | DCA | - | Nomogram |
| Wang J et al^[16]^ | 1,388/595 | AUC,Accuracy,Sensitivity,Specificity,PPV,NPV | 1 | LR | Yes,Random split validation | - | 47:(1)Demographic data; (2)vital signs and laboratory values; (3)Comorbidities; (4)Severity score; (5)Outcomes data. | Univariate logistic regression, stepwise regression | Multiple imputation | Yes, Calibration curve | DCA | - | Nomogram |
| Taylor RA et al^[17]^ | 4,222/1,056 | AUC | 3 | RF,LR,CART | Yes,Random split validation | - | (1)Demographic information; (2)previous health status; (3)ED health status; (4)ED services rendered; (5)Perational details. | Univariate | Missiong values were processed within models | - | - | - | - |
| Seo MH et al^[18]^ | 393/168 | AUC | 1 | LR | Yes,Random split validation | - | 21 :(1)Demographic data; (2)Pre-existing chronic comorbidities; (3)Initial vital signs; (4)Laboratory data. | Univariate logistic regression | - | Yes, Hosmer-Lemeshow test | - | - | Nomogram |
| Zhao C et al^[19]^ | 3,964/1,699 | AUC | 1 | RF | Yes,Random split validation | - | 20:(1) Demographics; (2) severity of illness; (3) Comorbidity conditions; (4) Interventions recieved in the first 24h; (5)Vital signs. | Univariate and multivariate logistic regression | - | Yes,Calibration curve | DCA | - | Nomogram |
| Zhang K et al^[20]^ | 5,443/- | AUC | 4 | LR,MARS,RF,XGBoost | - | Yes | 35 :(1)Demographic information; (2) ICU details; (3)Traditional severity scores. | Lasso | Mean imputation | Yes, Brier score | DCA | - | - |
| Zeng Z et al^[21]^ | 12,558/- | AUC,Accuracy,F1 score | 1 | blending model(LR\LDA\CART\NB\KNN\MLP\SVM\RF\XGB) | - | Yes | 65:(1)Demographics; (2)Vital signs; (3)Laboratory findings; (4)Comorbidities; (5)Pivotal treatments. | Stepwise logistic regression | KNN imputation | Yes, Calibration curve | - | - | - |
| Zeng Q et al^[22]^ | 161/70 | AUC | 1 | RF | Yes,Random split validation | - | 31:(1)Demographics;(2)Comorbidity;(3)Source of infection; (4)Laboratory data. | Multivariate logistic regression, stepwise algorithm | - | Yes,Calibration plots and Hosmer-Lemeshow test | DCA | - | Nomogram |
| Wernly B et al^[23]^ | 9,605/3,979 | AUC,PPV,NPV | 2 | LSTM,LR | Yes,Random split validation,cross-validation | - | Laboratory finding. | Univariable and multivariable logistic regression | Forward filling strategy | - | - | - | - |
| Wang H et al^[24]^ | 4,048/1,735 | AUC,Accuracy,Specificity,F1 score | 16 | Ridge classifier, Perceptron, Passive-Aggressive, kNN, Random Forest, Linear SVC (L1-L2), SGD (L1-L2-EN), MultinomialNB,BernoulliNB,Logistic Regression, SVM (rbf-poly-sigmoid) | Yes,Random split validation | - | 5:Race, sex, marital status, insurance types and languages. | Predefined | Deletion | - | - | - | - |
| van Doorn WPTM et al^[25]^ | 1,244/100 | AUC,Accuracy,Sensitivity,Specificity,PPV,NPV | 1 | XGBoost | Yes,5-fold cross-validation | - | - | Univariate and multivariate logistic regression | Machine learning model | Yes,Calibration curve and brier score | - | SHAP | - |
| Su L et al^[26]^ | 1,557/667 | AUC,Sensitivity,Specificity,F1 score | 3 | LR , RF , XGBoost | Yes,5-fold cross validation | - | 25: (1)Demographic data; (2)vital signs and laboratory values; (3)Traditional severity scores. | LASSO | KNN imputation | - | - | - | - |
| Rodríguez A et al^[27]^ | 2,008/502 | Accuracy,AUC | 4 | DT, RF, ANN , SVM | Yes,Random split validation, 10-fold cross-validation | - | 13:Age, SBP, Charlson index, PaO2-FiO2, platelets, GCS, cardiovascular score according to MAP ≥ 70, MAP <70 or onset of vasopressor, creatinine, temperature, RR, pH, hematocrit and lactate value | - | Deletion | - | - | - | - |
| Perng JW et al^[28]^ | 29,554/12,666 | AUC,Accuracy | 4 | RF, KNN, SVM, Softmax | Yes,K-fold cross validation | - | 53: (1)Demographic data; (2)vital signs and laboratory values. | CNN | Mean or median replacement,construction of an L1 or L2 constraint in the neural network | - | - | - | - |
| Park JY et al^[29]^ | 704,246/219,513 | AUC,Sensitivity,Spesificity,PPV,NPV | 5 | LASSO, RF Xgboost, DNN,Super Learner | Yes,Random split validation | - | 50:Demographics, Comorbidities, Hospital diagnosis, Measures of acute illness severity, Insurance, teaching status of hospital, System dysfunction, Tracheostomy. | RF | Deletion | Yes,Brier score | - | SHAP | - |
| Liu N et al^[30]^ | 308/34 | AUC | 1 | LR | Yes,10-fold cross-validation | - | 87: (1)Demographics; (2) Vital signs; (3)Medical history; (4)Laboratory investigations. | Backward stepwise multivariate logistic regression | Median imputation | - | - | - | - |
| Liu H et al^[31]^ | 3,667/1,573 | AUC | 1 | COX | Yes,Random split validation | - | 28:(1)Demographics;(2) Traditional severity scores; (3)Comorbidities; (4)Laboratory tests. | Cox regression,backward stepwise selection | multiple difference complement method | Yes,Calibration plots | DCA | - | Nomogram |
| Li K et al^[32]^ | -/- | AUC,Accuracy, F1 score | 5 | GBDT, LR, KNN, RF, SVM | Yes,5-fold cross-validation | - | (1)Demographics; (2)length of hospital stay; (3)vital signs and laboratory values; | DT | Mean imputation | - | - | - | - |
| Lagu T et al^[33]^ | 166,931/- | AUC | 1 | LR | - | Yes | (1)Demographics; (2)Site and type of infection; (3) Comorbidities; (4)Early treatment. | - | - | Yes,Hosmer-Lemeshow test and calibration plots | - | - | Logistic regression equation |
| Kong G et al^[34]^ | -/- | AUC,Sensitivity,Specificity | 4 | LASSO, RF, GBM, LR | Yes,5-fold cross validation | - | 86: (1)Demographics; (2)Laboratory tests; (3) Comorbidities. | - | Mean imputation | Yes,Calibration plots | - | - | - |
| Karlsson A et al^[35]^ | 356/89 | AUC, Sensitivity, Specificity, PPV, NPV | 1 | RF | Yes,10-fold cross validation | - | 91: (1)Vital signs; (2)Symptoms; (3)Observations and information from medical history. | Exclusion of the least important variable in Gini Impurity | Deletion | - | - | SHAP | - |
| Hu C et al^[36]^ | 7,053/1,764 | AUC,Accuracy | 7 | SVM,KNN, XGBoost, DT, NB, RF,LR | Yes,Random split validation | - | 57: (1)Demographic; (2)Comorbidities; (3)Vital signs; (4)Laboratory findings; (5)Medical treatments; (6)Urine output, (7)GCS. | LASSO | Multiple imputation by chained equations | Yes,Calibration plots | - | SHAP | - |
| Hou N et al^[37]^ | 4,559/- | AUC | 2 | XGBoost,LR | - | - | - | Backward stepwise logistic regression | Median imputation | - | DCA | - | Nomogram |
| Hargovan S et al^[38]^ | -/2,000 | AUC,Sensitivity,Specificity | 1 | LR | Yes,Simple sampling method | - | 106:- | Multivariable backward stepwise logistic regression | Deletion | Yes,Hosmer-Lemeshow test | - | - | - |
| García-Gallo JE et al^[39]^ | 3,955/1,695 | AUC | 1 | SGB | Yes,Random split validation | - | 140: (1)Laboratory measurements; (2)Routine charted data; (3)Comorbidities; (4)Organ dysfunction. | LASSO, SGB variable importance | Missing values were precossed within models | Yes, Hosmer–Lemeshow test | - | - | - |
| Ford DW et al^[40]^ | 65,069/433,379 | AUC | 1 | LR | Yes,Random split validation | Yes | 33: (1)Demographic; (2)Measures of acute illness severity; (3)Comorbidities. | Univariate | - | Yes,Hosmer-Lemeshow test | - | - | - |
| Phillips GS et al^[41]^ | 38,884/4,319 | AUC | 1 | LR | YesRandom split validation | - | (1)Demographics; (2)Comorbidities; (3)Illness severity; (4)Outcome. | Multivariable logistic regression | Single imputation | Yes,Hosmer-Lemeshow test | **-** | - | **-** |
| Ribas Ripoll VJ et al^[42]^ | 320/80 | AUC,Accuracy,Sensitivity,Specificity | 1 | LR | Yes,10-fold cross-validation | - | SOFA and SAPS | - | No missing values | - | **-** | - | **-** |
| Gong M et al^[43]^ | 16,520/- | AUC,Sensitivity,Specificity | 5 | SVM, LR, XGBoost，LSTM,TCN | Yes,Five-fold crossvalidation | Yes | 4: Vital signs (MAP, HR, RR, SpO2) | - | Last observation carried forward | - | - | - | - |
| García-Gallo J E et al^[44]^ | 3,955/1,695 | AUC | 1 | BART | Yes,Random split validation | - | 20:(1)Demographic data; (2)vital signs and laboratory values; (3)Comorbidities; (4)GCS. | LASSO, Stochastic Gradient Boosting (SGB) | Missing values were precossed within models | Yes,Hosmer-Lemeshow test | - | - | - |
| Wang W et al^[45]^ | -/- | AUC,Accuracy,Sensitivity,Specificity | 1 | LR | - | - | 4:PCT,suPAR,APACHE II,SOFA | Univariate | Deletion | - | - | - | Logistic regression equation |
| Ding X et al^[46]^ | 96/- | AUC, Accuracy, Sensitivity, Specificity | 1 | LR | - | - | Metabolites + clinical indicators | Univariate | Deletion | - | - | - | - |
| Wang L et al^[47]^ | 175/- | AUC, Sensitivity, Specificity, PPV, NPV | 1 | LR | - | - | 8:suPAR, qSOFA, lactate, CRP, PCT, SOFA, APACHE II, comorbidities | Univariable + multivariable logistic regression | - | - | - | - | - |
| Cheng YW et al^[48]^ | 15,760/3,383 | AUC, Sensitivity, Specificity, PPV, NPV, F1 score | 1 | XGBoost | Yes,non random time split validation | - | 39: (1)Demographics; (2)Vital signs; (3)GCS; (4)Labs; (5)Comorbidities; (6)Infection site; (7)Treatment; (8)SOFA, APACHE II. | XGBoost+Variable importance ranking | Last observation carried forward | Yes,Hosmer-Lemeshow test and calibration curves | - | SHAP | - |
| Zhuang J et al^[49]^ | 16,741/- | AUC | 1 | XGBoost | Yes,Nested and -n-nested cross-validation (8-fold cross validation) | Yes | 15: (1)Demographic; (2)SOFA; (3)Vital signs; (4)Laboratory findings;(5)Urine output | SHAP value ranking + physician consensus | Median imputation | Yes,calibration curves+Brier score | DCA | SHAP | - |
| Zheng F et al^[50]^ | 9,558/2,389 | AUC | 1 | XGBoost（ShockSurv） | Yes,Random split validation and 5-fold crossvalidation | Yes | 88:- | Variables with missing rate >30% were removed, except ALT, AST, albumin, bilirubin due to clinical significance | Multiple imputation | Yes,Calibration curve | DCA | SHAP | - |
| Pan X et al^[51]^ | 16,005/7,884 | AUC,Accuracy | 3 | LR, GNB, SVM | Yes,Random split validation, 5-fold cross-validation | - | 6:Six SOFA component scores | Logistic regression+ SelectFromModel | KNN imputation | - | DCA | - | - |
| Li S et al^[52]^ | 16,208/4,013 | AUC,Accuracy,Sensitivity,Specificity,PPV,NPV | 2 | XGBoost,LR | Yes,Random split validation,5-fold crossvalidation | - | 125: (1)Demographic; (2)Comorbidities; (3)Vital signs; (4)Laboratory findings; (5)Ventilation, (6)Renal replacement therapy;(7)Vasopressors; (8)Nutrition; (9)Urine output (10)GCS. | All included | Multiple imputation | Yes,Calibration plots | - | SHAP | - |
| Bao C et al^[53]^ | 12,861/- | AUC, F1 score | 7 | SVM, DT, RF, GBM, MLP, XGBoost, LightGBM | Yes,5-fold crossvalidation | Yes | (1)Demographic; (2)Vital signs; (3)Laboratory findings; (4)GCS; (5)Urine output. | LASSO | Variables >20% missing removed; <20% imputed with MICE | Yes,Calibration plots | DCA | - | - |
| Wang ZY et al^[54]^ | 12,664/12,664 | AUC,Accuracy,F1 score | 5 | LightGBM, XGBoost, RF, LR, BN | Yes,10-fold cross validation | Yes | 19: (1)Laboratory findings; (2)Antibiotics; (3)Charlson comorbidity score; (4)ICU and hospital LOS; (5)Vital signs; (6)Urine output, (7)Ventilation; (8)Specimen. | Maximal Information Coefficient (MIC) + grid search | >10% deleted, <10% filled with mode | - | - | SHAP | - |
| Su Y et al^[55]^ | 1,698/1,176 | AUC, Accuracy, Sensitivity, Specificity | 2 | ANN, LR | Yes,5 fold cross-validation + Random split validation | - | 11: (1)Demographic; (2)Laboratory findings. | Multivariable Logistic regression | Multiple imputation | - | - | SHAP | - |
| Ke X et al^[56]^ | 14,818/3,704 | AUROC, Accuracy, F1 score | 6 | XGBoost, LightGBM, LR, RF, DT, KNN | Yes,Random split validation,10-fold crossvalidation | - | 59: (1)Demographic; (2)Vital signs; (3)Laboratory findings; (4)Mechanical ventilation; (5)Renal replacement therapy; (6)Comorbidities; (7) Traditional severity scores; (8) Urine output. | Clinical significance + SHAP feature importance | KNN imputation | - | - | SHAP | - |
| Zhang Z et al^[57]^ | 2,501/705 | AUC | 1 | LASSO | Yes,Random split validation | - | 65: (1)Demographics; (2)Laboratory findings; (3)Vital signs; (4)Comorbidities; (5)Vasopressors; (6)GCS; (7)Urine output. | LASSO | Deletion | Yes,Calibration curve and Brier score | - | - | - |
| Wang M et al^[58]^ | 878/375 | AUC | 6 | LR, RF, SVM, XGBoost, DT, Ensemble model | Yes,Split-cohort internal validation | - | 8: (1)Demographic; (2) Laboratory findings | Multivariable logistic regression | Multiple imputation | Yes,calibration curves | DCA | - | Nomogram |
| Chicco D et al^[59]^ | 110,204/19,051 | AUC,Accuracy,PPV,NPV,F1 score | 5 | Radial SVM,GB,NB,LR,Linear SVM | Yes,Random split validation | Yes | 3: Demographics (Sex, age) and septic episode number | Predefined | No imputation needed | - | - | - | - |
| Adrie C et al^[60]^ | 1,458/810 | AUC | 1 | LR | Yes,Random split validation | - | LOD, SOFA, SAPS II, septic shock, episode number, multiple sites, McCabe score, chronic illness, infection site, pathogen type, ventilation, catheter, renal replacement, early antibiotics, etc. | Univariable | - | Yes,Hosmer-Lemeshow test and calibration curves | - | - | Logistic regression equation |
| Chen SH et al^[61]^ | 739/- | AUC | 1 | Cox proportional hazard model | - | - | (1)Demographic data; (2)Comorbidities; (3) Infection sites; (4)Labs; (5) Traditional severity scores. | Cox regression | Deletion | Yes,Calibration plots | - | - | Nomogram |
| Cheng CY et al^[62]^ | 116,188/116,188 | AUC,Accuracy,Sensitivity | 3 | CNN, LSTM , RF | Yes,5-fold cross-validation | Yes | 7:Age, sex, SBP, DBP, HR, RR, BT | All included | Forward-fill imputation | - | - | - | - |
| Gao J et al^[63]^ | 5,843/- | AUC,Accuracy,Sensitivity,F1 score | 7 | DT, RF, GB, XGBoost, LightGBM, SVM, MLP | Yes,5-fold cross-validation | - | 38: (1)Demographic information; Vantibiotic usage; (3)Patient medical history; (4)Various laboratory results. | Literature review + clinical expert recommendation + statistical validation | - | - | - | SHAP | - |
| Greco, M et al^[64]^ | 297/128 | AUC,Accuracy,F1 score | 2 | LR, RF | Yes,10-fold cross-validation | - | (1)Labs; (2)Vitals; (3)Scores; (4)Meds, (5)Infection site. | Literature + clinical expertise + univariate analysis | Random forest imputation | - | - | - | - |
| He B et al^[65]^ | 4,375/1,459 | AUC, Accuracy, Sensitivity, Specificity, PPV, NPV, F1 score | 4 | XGBoost, RF, LR, SVM | Yes,5-fold cross-validation | - | 41: (1)Demographic characteristics; (2)Comorbidities; (3)Vital signs; (4)Laboratory results and severity scores | LASSO | Imputed by MICE | Yes, Hosmer-Lemeshow , Brier score and calibration curve | - | SHAP | - |
| Hong C et al^[66]^ | 1,300/433 | AUC, Accuracy, Sensitivity, Specificity | 1 | LR | Yes,Random split validation | - | 38:- | LASSO | Multiple imputation (MICE) | Yes,calibration plots | DCA | - | - |
| Jeon E et al^[67]^ | 607/203 | AUC | 5 | XGBoost, SVM, LightGBM, MLP,LR | Yes,Leave-one-out cross-validation strategy | - | 33: (1)Demographics; (2)Vitals; (3)Labs; (4)SOFA; (5)Infection source | RFE | Imputed by MICE | Yes,calibration curves + Brier score | - | SHAP | - |
| Jiang Z et al^[68]^ | 1,736/744 | AUC | 2 | XGBoost,LR | Yes,5-fold cross-validation | - | 188: (1)Demographics; (2)Vital signs (3)GCS; (4)Labs and ABGs | - | Mean imputation | - | - | SHAP | - |
| Koozi H et al^[69]^ | 1,984/- | AUC | 1 | LR | Yes,5-fold cross-validation | - | 36:- | Multivariate logistic regression | - | - | - | - | - |
| Li M et al^[70]^ | 545/- | AUC | 1 | LR | -- | - | (1)Demographic;(2)Clinical history; (3)Laboratory test data; (4)SOFA and APACHE II. | Multivariate logistic regression | - | Yes,Hosmer-Lemeshow test | - | - | Nomogram |
| Li Y et al^[71]^ | 274/108 | AUC, Sensitivity, Specificity, PPV, NPV | 1 | LR | Yes,Random split validation | - | (1)Demographic data; (2)Vital signs and laboratory values; (3)Comorbidities; (4)Outcomes data. | Multivariate logistic regression | - | Yes,Hosmer-Lemeshow test | - | - | - |
| Lin XM et al^[72]^ | 84/28 | AUC, Sensitivity, Specificity | 1 | LR | Yes,Random split validation | - | (1)Demographic characteristics; (2) Comorbidities; (3)Laboratory results | Multivariate logistic regression | - | - | - | - | - |
| Liu Y et al^[73]^ | 84/36 | AUC, Sensitivity, Specificity, PPV, NPV | 3 | LR, RF,LR+RF | Yes,Random split validation | - | (1)Basic data; (2)Serological parameter; (3) Scale score | Multivariate logistic regression | Mean imputation | - | - | - | - |
| Lu B et al^[74]^ | 1,218/522 | AUC, Sensitivity, Specificity | 1 | LR | Yes,Random split validation | - | 24:(1)Demographic data; (2) Biochemical markers; (3)Clinical indicator. | Multivariate logistic regression | Multiple imputation | Yes,Calibration curve | DCA | - | Nomogram |
| Park SW et al^[75]^ | 4,089/- | AUC, Specificity, F1 score | 6 | LR, SVM, RF, XGBoost, LightGBM, CatBoost | Yes,5-fold cross-validation | - | 44: (1)Demographic characteristics; (2)Clinical information; (3)Comorbidities; (4)Laboratory findings; (5)characteristics of infection | Variables with <10% missing selected by specialist | Deletion | - | - | SHAP | - |
| Pérez-Tome JC et al^[76]^ | 144/36 | AUC,Accuracy,Sensitivity, Specificity | 1 | RF | Yes,Random split validation | Yes | 43: (1)Demographic data; (2)Vital signs; (3)Laboratory values; (4)Comorbidities; (4)Severity score; (5)Outcomes data | RF feature importance | - | - | - | - | - |
| Rahman MS et al^[77]^ | 3,392/848 | AUC, Accuracy, Specificity, F1 score | 8 | LR, RF, ET, XGBoost, CatBoost, AdaBoost, GB, MLP | Yes,5 fold cross-validation | - | 15:- | - | MICE imputation | Yes,calibration curve | DCA | SHAP | Nomogram |
| Wang B et al^[78]^ | 844/361 | AUC,Accuracy, Sensitivity, Specificity, PPV, NPV | 1 | LR | Yes,Random split validation | - | (1)Demographic information; (2)GCS; (3)MAP;(4)Hematological indices | Multivariate logistic regression | Deletion | Yes,Calibration plots | DCA | - | Nomogram |
| Selcuk M et al^[79]^ | 160/- | AUC, Sensitivity, Specificity | 8 | MLP, LR, RF, SVM, XGB, KNN, DT, GNB | Yes,Random split validation+10-fold cross-validation | - | 38: (1)Demographics; (2)Vital signs;  (3)Laboratory findings; (4)ICU Admission reason; (5)Vasopressor; (6)Comorbidities; (7)Severe organ system insufficiency;(8)Mechanical ventilation; (7)Urine output. | All variables required by APACHE II, SAPS II, SOFA | No missing values | - | - | - | - |
| Xie Y et al^[80]^ | 257/110 | AUC | 1 | LR | Yes,Random split validation | - | (1)Demographic data; (2)Comorbidities; (3)Mechanical ventilation;(4) MAP;(5)vasopressor use; (6)type and source of infection;(7)days of hospital and ICU stay | Multivariate logistic regression | Multiple imputation | Yes,Calibration plot | DCA | - | Nomogram |
| Zheng YJ et al^[81]^ | 801/534 | AUC | 1 | LR | Yes,Random split validation | - | 45: (1)Demographic; (2)Clinical; (3)Laboratory data. | Multivariable logistic regression | Deletion | Yes,Hosmer-Lemeshow test | - | - | - |
| Wang S et al^[82]^ | 2,760/691 | AUC, Accuracy, Sensitivity, Specificity, F1 score | 1 | TCF | Yes,5-fold cross-validation + 1000 bootstrap resampling | Yes | 93:- | Logistic Regression,entropy-based ranking | Multiple Imputation | Yes,Calibration curve | DCA | SHAP | - |
| Chen T et al^[83]^ | 840/168 | AUC,Accuracy,Sensitivity,Specificity, F1 score | 4 | LR,SVM,XGBoost,SWSELM | Yes,5-fold cross-validation | - | 27:Oxygenation Index; PCT; PT; Gender; Age; Onset Time; BMI; NT-proBNP; Infection Site; MAP; HR; PO2; Urine pH; Lactate; APTT; CRP; pH; Diabetes; Platelets; Carbon Dioxide Content; Charlson Comorbidity Index;RR; Albumin; Oxygen Saturation; HR-SBP; Hematocrit; Creatinine. | LASSO+Multivariate logistic regression | Median for continuous, Mode for categorical | - | - | SHAP | - |
| Kurtkulagi O et al^[84]^ | -/- | AUC | 1 | LR | - | - | (1)Demographic information;(2)Comorbidities;(3)Functional and physical status; (4)Sources of infection; (5)Lactate; (6)Mechanical ventilation status;(7)final diagnoses. | Stepwise multivariate logistic regression | Deletion | Yes,Hosmer–Lemeshow test | - | - | - |
| Li Q et al^[85]^ | 1,116/116 | AUC,Sensitivity,Specificity | 1 | Cox | Yes,Bootstrap resampling (1000 iterations) | - | (1)Demographic information;(2)Laboratory data. | Cox regression | Deletion | - | - | - | Nomogram |
| Shi S et al^[86]^ | 8,265/3,534 | AUC | 9 | LR,GNB,KNN,SVM,ANN,DT,RF,GBM,CatBoost | Yes,5-fold cross-validation | Yes | 31: (1)Demographic data;(2)Laboratory indicators. | Multivariable logistic regression | KNN imputation | Yes,Calibration curves | DCA | SHAP | - |
| Shi W et al^[87]^ | 1,673/716 | AUC | 3 | Cox | Yes,Random split validation | - | 35: (1)Demographics; (2)Comorbidities; (3)Infection source-pathogen; (4)Laboratory indicators; (5)SOFA, APACHE II; (6)Interventions | LASSO + Cox regression | >20% missing excluded; cross-validation and residual analysis | - | DCA | - | Nomogram + online calculator |
| Wang Y et al^[88]^ | 19,000/8,134 | AUROC,Sensitivity,Specificity,Accuracy,PPV,NPV,F1 score | 4 | XGBoost,LR,SVM,DNN | Yes,Random split validation | Yes | 52: (1)Demographics; (2)Vitals; (3)Labs, (4)Treatments; (5) severity scores. | - | Multiple imputation | Yes,Calibration curves | DCA | SHAP | - |
| Yang Y et al^[89]^ | 580/580 | AUC | 1 | LR | Yes,Random split validation | Yes | 58: (1)Demographics; (2)Labs; (3)Vitals; (4)Treatments; (5)SOFA, APACHEII. | Multivariate logistic regression | Multiple imputation | Yes,Hosmer–Lemeshow test and calibration curves | DCA | SHAP | Nomogram |
| Zhang Y et al^[90]^ | 180/180 | AUC, Sensitivity, Specificity | 1 | LR | Yes,Bootstrap resampling | - | 38: (1)Demographics; (2)Vital signs; (3)laboratory parameters;(4)disease-related scores;(5)major comorbidities. | Univariate + LASSO | - | Yes,Calibration curves | DCA | - | Nomogram |
| Zhu XY et al^[91]^ | 2,865/1,191 | AUC,Accuracy,Sensitivity,Specificity,PPV,NPV,F1 score | 6 | LR, NN, SVM, MLP, Naïve Bayes, XGBoost | Yes,Random split validation + 10-fold cross-validation | - | 49: (1)Demographics; (2)Vitals; (3)Labs; (4)Comorbidities | RFE | >30% missing excluded,others Multiple imputation | Yes,Calibration curves | DCA | SHAP | - |

Abbreviations: AUC, Area Under the Receiver Operating Characteristics; PPV, Positive predictive value; NPV, Negative predictive value; LR, logistic regression model; RF, Random Forest; XGBoost, eXtreme Gradient Boosting; KNN, K-nearest neighbor; NB, Naive Bayes; SVM, Support vector machine; CART, Classification And Regression Tree; MARS: Multivariate Adaptive Regression Splines; ANN, Artificial neural network; GBM, Gradient boosting Machine; TCN, Temporal convolutional network; LSTM, Long short-term memory; Cox, cox proportional regression hazards model; GBDT, Gradient boosting decision tree; DNN, Deep Neural Network, GCN, Graph Convolutional Network; DT, Decision tree; MLP, Multi-layer perceptron neural network; GCS, glasgow coma scale; DCA: Decision curve analysis; LASSO, Least Absolute Shrinkage and Selection Operator; SHAP, the SHapley Additive exPlanations; -, not reported.
