# Supplementary material for: Prediction models for mortality in patients with sepsis: a systematic review and meta-analysis
Source: Front Med (Lausanne). 2026 Jun 10;13:1730156. doi: 10.3389/fmed.2026.1730156 (PMC13290529; doi:10.3389/fmed.2026.1730156)
Supplement: Supplementary file 4 [file Table_4.DOC]

**Supplementary Table 2**

**Details information on the development model and the internal validation of the model performance**

| **Author** | **AUC（D/I）** | **Accuracy**  **（D/I）** | **Sensitivity**  **（D/I）** | **Specificity（D/I）** | **PPV（D/I）** | **NPV（D/I）** | **F1 score（D/I）** | **AUC of severity scores** |
| --- | --- | --- | --- | --- | --- | --- | --- | --- |
| Zhi D et al^[8]^ | RF(0.754/-), LR(0.703/-) | - | RF(0.923/-), LR(0.633/-) | RF(0.307/-), LR(0.664/-) | RF(0.837/-), LR(0.593/-) | RF(0.508/-), LR(0.700/-) | RF(0.539/-) | - |
| Zhang G et al^[9]^ | XGboost(0.94/0.771), LR(0.707/0.703), RF(0.686/0.667), KNN(0.622/0.617), NB(0.590/0.690), SVM(0.648/0.658), DT(0.595/0.60) | XGboost(0.882/0.846), LR(0.850/0.836), RF(0.852/0.832), KNN(0.855/0.786), NB(0.842/0.828), SVM(0.855/0.830), DT(0.853/0.790) | - | - | - | - | XGboost(0.937/0.96),LR(0.915/0.90),RF(0.917/0.903),KNN(0.989/0.877),NB(0.914/0.909),SVM(0.892/0.896),DT(0.871/0.888) | SOFA:0.687 |
| Yu Z et al^[10]^ | LR(-/0.661), LightGBM(-/0.983), CatBoost(-/0.853), RF(-/0.926) , KNN(-/0.863) ,SVM(-/0.832) , XGBoost(-/0.840) | LR(-/0.846), LightGBM(-/0.966), CatBoost(-/0.936), RF(-/0.968) , KNN(-/0.840) ,SVM(-/0.837) , XGBoost(-/0.926) | - | - | - | - | LR(-/0.479), LightGBM(-/0.910),CatBoost(-/0.818), RF(-/0.915) , KNN(-/0.546) ,SVM(-/0.339) , XGBoost(-/0.790) | - |
| Li Y et al^[11]^ | - | Deep Learning model(-/0.8278) | - | - | - | - | - | - |
| Xu Z et al^[12]^ | LR(0.829/0.825) | LR(0.773/0.747) | LR(0.783/0.677) | LR(0.740/0.923) | LR(0.514/0.533) | LR(0.906/0.957) | - | SOFA:0.707(0.584-0.830);APACHE II:0.718(0.601-0.835) |
| Li F et al^[13]^ | LR(0.912/-) | - | - | - | - | - | - | - |
| García de Guadiana-Romualdo L et al^[14]^ | LR(0.766/-) | - | - | - | - | - | - | SOFA:0.736(0.664-0.800) |
| Li F et al^[15]^ | LR(0.83/-) | - | LR(0.804) | LR(0.807) | - | - | - | APACHE II:0.64 (0.59-0.69); SOFA： 0.83 (0.79-0.87) |
| Wang J et al^[16]^ | LR(0.773/0.750) | LR(0.718/0.691) | LR(0.761/0.751) | LR(0.662/0.619) | LR(0.747/0.703) | LR(0.679/0.673) | - | SOFA: 0.542 (0.495-0.588);SAPS II:0.653（0.609-0.697） |
| Taylor RA et al^[17]^ | RF(-/0.860), LR(-/0.755), CART(-/0.693) | - | - | - | - | - | - | - |
| Seo MH et al^[18]^ | LR(0.8173/0.7537) | - | - | - | - | - | - | APACHE II:0.6177 (0.5423-0.6931)；SOFA: 0.6005 (0.5256-0.6754) |
| Zhao C et al^[19]^ | RF(0.826/0.813) | - | - | - | - | - | - | SOFA:0.680(0.648-0.712);SAPS II:0.744（0.715-0.772） |
| Zhang K et al^[20]^ | LR(0.799/-),MARS(0.813/-),RF(0.816/-),XGBoost(0.895/-) | - | - | - | - | - | - | - |
| Zeng Z et al^[21]^ | - | - | - | - | - | - | - | SAPS II (0.77);SOFA (071) |
| Zeng Q et al^[22]^ | RF(0.810/0.813) | - | - | - | - | - | - | - |
| Wernly B et al^[23]^ | LSTM(0.88/0.85),LR(0.82/0.81) | - | - | - | LSTM(0.60/0.43),LR(0.48/0.35) | LSTM(0.96/0.96),LR(0.95/0.96) | - | SOFA (0.72) |
| Wang H et al^[24]^ | Ridge classifier(-/0.7774),Perceptron(-/0.7786),Passive-aggressive(-/0.7582),kNN(-/0.7299),RF(-/0.6459),LinearSVC_L1(-/0.7781),LinearSVC_L2(-/0.7777),SGDClassifier_L1(-/0.7759),SGDClassifier_L2(-/0.7749),SGDClassifier_EN(-/0.7753),MultinomialNB(-/0.7040),BernoulliNB(-/0.5724),LR(-/0.7761),SVC_rbf(-/0.7744),SVC_poly(-/0.7751),SVC_sigmoid(-/0.6873) | Ridge classifier(-/0.6790),Perceptron(-/0.6720),Passive-aggressive(-/0.6841),kNN(-/0.7135),RF(-/0.7516),LinearSVC_L1(-/0.6749),LinearSVC_L2(-/0.6784),SGDClassifier_L1(-/0.6790),SGDClassifier_L2(-/0.6790),SGDClassifier_EN(-/0.6801),MultinomialNB(-/0.6392),BernoulliNB(-/0.3107),LR(-/0.6842),SVC_rbf(-/0.6847),SVC_poly(-/0.6749),SVC_sigmoid(-/0.6277) | - | Ridge classifier(-/0.6745),Perceptron(-/0.6664),Passive-aggressive(-/0.6792),kNN(-/0.7305),RF(-/0.7999),LinearSVC_L1(-/0.6698),LinearSVC_L2(-/0.6739),SGDClassifier_L1(-/0.6745),SGDClassifier_L2(-/0.6759),SGDClassifier_EN(-/0.6765),MultinomialNB(-/0.6354),BernoulliNB(-/0.2042),LR(-/0.6772),SVC_rbf(-/0.6833),SVC_poly(-/0.6698),SVC_sigmoid(-/0.6159) | - | - | Ridge classifier(-/0.5855),Perceptron(-/0.5801),Passive-aggressive(-/0.5907),kNN(-/0.5981),RF(-/0.5991),LinearSVC_L1(-/0.5823),LinearSVC_L2(-/0.5850),SGDClassifier_L1(-/0.5855),SGDClassifier_L2(-/0.5843),SGDClassifier_EN(-/0.5858),MultinomialNB(-/0.5487),BernoulliNB(-/0.3096),LR(-/0.5893),SVC_rbf(-/0.5882),SVC_poly(-/0.5823),SVC_sigmoid(-/0.5451) | - |
| van Doorn WPTM et al^[25]^ | XGBoost(0.84/0.85) | XGBoost(-/0.80) | XGBoost(-/0.92) | XGBoost(-/0.78) | XGBoost(-/0.39) | XGBoost(-/0.96) | - | - |
| Su L et al^[26]^ | LR(-/0.73)+F:R,RF(-/0.74),XGBoost(-/0.71) | - | LR(-/0.65),RF(-/0.50),XGBoost(-/0.33) | LR(-/0.72),RF(-/0.84),XGBoost(-/0.89) | - | - | LR(-/0.62),RF(-/0.66),XGBoost(-/0.62) | SOFA(0.63);APACHE II (0.61) |
| Rodríguez A et al^[27]^ | DT(0.59/0.53), RF(0.79/0.65), SVM(0.82/0.69), ANN(0.77/0.69) | DT(0.59/0.64), RF(0.78/0.74), SVM(0.81/0.71), ANN(0.77/0.69) | - | - | - | - | - | - |
| Perng JW et al^[28]^ | 72h:RF(-/0.87), KNN(-/0.86), SVM(-/0.92), Softmax(-/0.94);28day:RF(-/0.90), KNN(-/0.86), SVM(-/0.92), Softmax(-/0.92) | 72h:RF(-/0.61), KNN(-/0.82), SVM(-/0.85), Softmax(-/0.87);28day:RF(-/0.61), KNN(-/0.82), SVM(-/0.85), Softmax(-/0.87) | - | - | - | - | - | qSOFA:0.68(0.67-0.69) |
| Park JY et al^[29]^ | LASSO(-/0.878), RF(-/0.878),XGboost(-/0.888), DNN(-/0.893),Super Learner(-/0.883) | - | LASSO(-/0.812),RF(-/0.818),XGboost(-/0.829),DNN(-/0.826),Super Learner(-/0.833) | LASSO(-/0.784),RF(-/0.771),XGboost(-/0.781), DNN(-/0.794),Super Learner(-/0.769) | LASSO(-/0.468), RF(-/0.454),XGboost(-/0.472), DNN(-/0.484),Super Learner(-/0.458) | LASSO(-/0.947),RF(-/0.9487),XGboost(-/0.952), DNN(-/0.951),Super Learner(-/0.952) | - | - |
| Liu N et al^[30]^ | LT(-/0.77) | - | - | - | - | - | - | SOFA:0.71;qSOFA:0.72;APACHE II：0.74 |
| Liu H et al^[31]^ | COX:30day(0.766/0.759);60day(0.771/0.770);90day:(0.772/0.760) | - | - | - | - | - | - | SOFA30day:0.700;SOFA60day:0.678;SOFA90day:0.662 |
| Li K et al^[32]^ | GBDT(-/0.992), LR(-/0.980), KNN(-/0.898), RF(-/0.877), SVM(-/0.876) | GBDT(-/0.954), LR(-/0.938), KNN(-/0.860), RF(-/0.819), SVM(-/0.821) | - | - | - | - | GBDT(-/0.933), LR(-/0.907), KNN(-/0.780), RF(-/0.702), SVM(-/0.715) | - |
| Lagu T et al^[33]^ | LR(0.78/-) | - | - | - | - | - | - | APACHE II: 0.712 (0.630-0.756)；SAPS II:0.653（0.681-0.803） |
| Kong G et al^[34]^ | LASSO(-/0.829), RF(-/0.845), GBM(-/0.829), LR(-/0.833) | - | LASSO(-/0.744), RF(-/0.771), GBM(-/0.765), LR(-/0.760) | LASSO(-/0.754), RF(-/0.755), GBM(-/0.740), LR(-/0.748) | - | - | - | SAPS II:0.77 |
| Karlsson A et al^[35]^ | RF:7day(-/0.83);30day(-/0.80) | - | RF:7day(-/0.84);30day(-/0.87) | RF:7day(-/0.67);30day(-/0.64) | RF:7day(-/0.31);30day(-/0.41) | RF:7day(-/0.96);30day(-/0.95) | - | - |
| Hu C et al^[36]^ | SVM(-/0.763),KNN(-/0.651), XGBoost(-/0.884), DT(-/0.655), NB(-/0.856), RF(-/0.882),LR(-/0.845) | SVM(-/0.872),KNN(-/0.868), XGBoost(-/0.895), DT(-/0.842), NB(-/0.862), RF(-/0.891),LR(-/0.890) | - | - | - | - | - | - |
| Hou N et al^[37]^ | XGBoost(0.819/-),LR(0.857/-) | - | - | - | - | - | - | SAPS II:0.80 |
| Hargovan S et al^[38]^ | LR(0.90/-) | - | LR(0.79/-) | LR(0.94/-) | LR(0.54/-) | LR(0.98/-) | - | APACHE II:0.69(0.59-0.79);SOFA:0.78(0.72-0.85);SAPS-II:0.81(0.75-0.87);qSOFA:0.60(0.50-0.70) |
| García-Gallo JE et al^[39]^ | SGB(-/0.8039) | - | - | - | - | - | - | - |
| Ford DW et al^[40]^ | LR(0.801/0.765) | - | - | - | - | - | - | - |
| Phillips GS et al^[41]^ | LR(0.770/0.773) | - | - | - | - | - | - | - |
| Ribas Ripoll VJ et al^[42]^ | LR (-/0.79) | LR (-/0.72) | LR (-/0.69) | LR (-/0.80) | - | - | - | SAPS:0.68 |
| Gong M et al^[43]^ | SVM(0.81/-), LR(0.78/-), XGBoost(0.83/-),LSTM(0.82/-),TCN(0.83/-) | - | SVM(0.59/-), LR(0.68/-), XGBoost(0.61/-),LSTM(0.68/-),TCN(0.68/-) | SVM(0.86/-), LR(0.75/-), XGBoost(0.88/-),LSTM(0.81/-),TCN(0.83/-) | - | - | - | - |
| García-Gallo J E et al^[44]^ | BART(0.7286/0.7348) | - | - | - | - | - | - | - |
| Wang W et al^[45]^ | LR(0.924/-) | LR(0.8247/-) | LR(0.942/-) | LR(0.932/-) | LR(0.891/-) | LR(0.65/-) | - | SOFA:0.756(0.736-0.8571);APACHE II：0.762(0.721-0.849) |
| Ding X et al^[46]^ | LR:28day(0.881);in-hospital(0.830);90day(0.886) | LR:28day(0.771);in-hospital(0.729);90day(0.823) | LR:28day(0.755);in-hospital(0.736);90day(0.833) | LR:28day(0.787);in-hospital(0.721);90day(0.786) | - | - | - | - |
| Wang L et al^[47]^ | LR(0.864/-) | - | LR(0.732/-) | LR(0.895/-) | LR(0.682/-) | LR(0.916/-) | - | qSOFA:0.832(0.692–0.923) |
| Cheng YW et al^[48]^ | XGBoost(-/0.853) | - | XGBoost(-/0.807) | XGBoost(-/0.743) | XGBoost(-/0.407) | XGBoost(-/0.946) | XGBoost(-/0.541) | SOFA(0.72);APACHE II (0.68) |
| Zhuang J et al^[49]^ | XGBoost(-/0.84) | XGBoost(-/0.75) | - | - | - | - | XGBoost(-/0.5) | SAPS II (0.77,EV:0.79);APS III (0.74,EV :0.86);APACHE II (EV: 0.83);SOFA(0.73, EV: 0.77, 0.71,0.51) |
| Zheng F et al^[50]^ | XGBoost(ShockSurv)(0.9161/0.9027) | - | - | - | - | - | - | - |
| Pan X et al^[51]^ | LR(0.76/0.757), GNB(0.76/0.762), SVM(0.67/0.630) | LR(0.851/-), GNB(0.844/-), SVM(0.862/-) | - | - | - | - | - | - |
| Li S et al^[52]^ | XGBoost(0.97/0.85),LR(0.84/0.82) | XGBoost(0.92/0.77),LR(0.73/0.70) | XGBoost(0.86/0.76),LR(0.81/0.80) | XGBoost(0.92/0.73),LR(0.72/0.67) | XGBoost(0.66/0.41),LR(0.33/0.38) | XGBoost(0.98/0.93),LR(0.96/0.93) | - | APS III (0.74);SAPS II (0.73);SOFA (0.63) |
| Bao C et al^[53]^ | SVM(0.70/0.75), DT(0.75/0.75), RF(0.82/0.81), GBM(0.85/0.85), MLP(0.82/0.82), XGBoost(0.84/0.84), LightGBM(0.86/0.85) | - | - | - | - | - | SVM(0.65/0.65), DT(0.79/0.80), RF(0.89/0.89), GBM(0.90/0.90), MLP(0.64/0.63), XGBoost(0.90/0.89),LightGBM(0.91/0.91) | - |
| Wang ZY et al^[54]^ | LightGBM(0.900/-), XGBoost(0.875/-), RF(0.891/-), LR(0.807/-), BN(0.756/-) | LightGBM(0.808/-), XGBoost(0.795/-), RF(0.806/-), LR(0.751/-), BN(0.654/-) | - | - | - | - | LightGBM(0.668/-), XGBoost(0.645/-), RF(0.657/-), LR(0.571/-), BN(0.507/-) | SAPS II (0.75) |
| Su Y et al^[55]^ | ANN(0.873/0.811), LR(0.720/0.752) | ANN(0.866/0.735), LR(0.711/0.722) | ANN(0.850/0.624),LR(0.662/0.604) | ANN(0.410/0.772),LR(0.337/0.744) | - | - | - | SOFA (0.70) |
| Ke X et al^[56]^ | XGBoost(-/0.871), LightGBM(-/0.870), LR(-/0.857), RF(-/0.807), DT(-/0.738), KNN(-/0.744) | XGBoost(-/0.851), LightGBM(-/0.876), LR(-/0.877), RF(-/0.866), DT(-/0.850), KNN(-/0.857) | - | - | - | - | XGBoost(-/0.547),LightGBM(-/0.467), LR(-/0.480), RF(-/0.362), DT(-/0.369), KNN(-/0.283) | - |
| Zhang Z et al^[57]^ | LASSO(-/0.772) | - | - | - | - | - | - | SAPS II (0.74);APS III (0.74);SOFA (0.69);qSOFA (0.54) |
| Wang M et al^[58]^ | LR(0.881/0.868),RF(-/0.865), SVM(-/0.837), XGBoost(-/0.834), DT(-/0.835), Ensemble model(-/0.863) | - | - | - | - | - | - | SOFA(0.79) |
| Chicco D et al^[59]^ | Radial SVM(0.966/0.858),GB(0.966/0.856),NB(0.954/0.841),LR(0.941/0.801),Linear SVM(0.860/0.860) | Radial SVM(0.515/0.467), GB(0.851/0.718), NB(0.567/0.465), LR(0.801/0.679), Linear SVM(0.337/0.337) | - | - | Radial SVM(0.970/0.861), GB(0.934/0.822), NB(0.965/0.861), LR(0.943/0.828), Linear SVM(0.896/0.896) | Radial SVM(0.112/0.222), GB(0.126/0.231), NB(0.117/0.220),LR(0.149/0.239), Linear SVM(0.210/0.210) | Radial SVM(0.652/0.553), GB(0.916/0.819), NB(0.703/0.551), LR(0.886/0.794), Linear SVM(0.333/0.333) | - |
| Adrie C et al^[60]^ | LR(0.822/0.763) | - | - | - | - | - | - | APACHE II：0.73;SAP II：0.72 |
| Chen SH et al^[61]^ | COX(0.65/-) | - | - | - | - | - | - | SOFA:0.652;qSOFA:0.536;APACHE II:0.592 |
| Cheng CY et al^[62]^ | CNN(0.82/0.83), LSTM(0.76/0.74), RF(0.78/0.77) | CNN(0.828/0.808), LSTM(0.759/0.733), RF(0.776/0.765) | CNN(0.854/0.718),LSTM(0.778/0.687),RF(0.737/0.692) | - | - | - | - | - |
| Gao J et al^[63]^ | SVM(-/0.76), DT(-/0.76), RF(-/0.93), GB(-/0.91), XGBoost(-/0.92), MLP(-/0.89), LightGBM(-/0.92) | SVM(-/0.665), DT(-/0.733), RF(-/0.863), GB(-/0.813), XGBoost(-/0.840), MLP(-/0.780), LightGBM(-/0.831) | SVM(-/0.618), DT(-/0.748), RF(-/0.837), GB(-/0.759), XGBoost(-/0.823),MLP(-/0.835),LightGBM(-/0.797) | - | - | - | SVM(-/0.646), DT(-/0.734), RF(-/0.857), GB(-/0.801), XGBoost(-/0.836), MLP(-/0.789), LightGBM(-/0.824) | - |
| Greco, M et al^[64]^ | LR(0.881/0.811), RF(0.923/0.863) | LR(0.886/0.773), RF(0.795/0.766) | - | - | - | - | LR(0.932/0.856), RF(0.866/0.847) | SOFA:0.712; APACHE II:0.664; qSOFA:0.706 |
| He B et al^[65]^ | XGBoost(0.888/0.806), RF(0.878/0.794), LR(0.836/0.782), SVM(0.732/0.687) | XGBoost(-/0.855), RF(-/0.846), LR(-/0.843), SVM(-/0.832) | XGBoost(-/0.761), RF(-/0.763), LR(-/0.732), SVM(-/0.621) | XGBoost(-/0.701), RF(-/0.690), LR(-/0.695), SVM(-/0.616) | XGBoost(-/0.577), RF(-/0.188), LR(-/0.442), SVM(-/0.216) | XGBoost(-/0.864), RF(-/0.873), LR(-/0.881), SVM(-/0.913) | XGBoost(-/0.373), RF(-/0.291), LR(-/0.429), SVM(-/0.271) | - |
| Hong C et al^[66]^ | LR(0.801/0.791) | LR(-/0.819) | LR(/0.348) | LR(-/0.953) | - | - | - | - |
| Jeon E et al^[67]^ | LightGB(-/0.87),XGBoost(-/0.84),SVM(-/0.85),MLP(-/0.86),LR(-/0.81) | - | - | - | - | - | - | qSOFA: 0.57(0.50-0.64);SOFA: 0.66(0.58-0.74) |
| Jiang Z et al^[68]^ | XGBoost(-/0.800),LR(-/0.756) | - | - | - | - | - | - | - |
| Koozi H et al^[69]^ | LR(0.72/0.71) | - | - | - | - | - | - | SAP III:0.75;SOFA:0.67 |
| Li M et al^[70]^ | LR(0.7207/-) | - | - | - | - | - | - | - |
| Li Y et al^[71]^ | LR(0.880/0.968) | - | LR(0.8115/-) | LR(0.8026/-) | LR(0.7995/-) | LR(0.8651/-) | - | - |
| Lin XM et al^[72]^ | LR(0.83/0.97) | - | LR(0.765/-) | LR(0.808/-) | - | - | - | SOFA:0.83(0.75-0.90);APACHE II:0.78(0.79-0.87);qSOFA:0.71(0.61-0.82) |
| Liu Y et al^[73]^ | LR(-/0.83),RF(-/0.81),LR+RF(-/0.92) | - | LR(-/0.800),RF(-/0.675),LR+RF(-/0.850) | LR(-/0.825),RF(-/0.813),LR+RF(-/0.917) | LR(-/0.696),RF(-/0.643),LR+RF(-/0.708) | LR(-/0.892),RF(-/0.833),LR+RF(-/0.963) | - | APACHE II:0.80, SOFA:0.71 |
| Lu B et al^[74]^ | LR(0.847/0.826) | - | LR(0.816/0.795) | LR(0.728/0.747) | - | - | - | SOFA:0.777(0.729-0.825) |
| Park SW et al^[75]^ | LR(-/0.772), SVM(-/0.771), RF(-/0.736), XGBoost(-/0.797), LightGBM(-/0.795), CatBoost(-/0.800) | - | - | LR(-/0.757), SVM(-/0.743), RF(-/0.747), XGBoost(-/0.929),LightGBM(-/0.861),CatBoost(-/0.91) | - | - | LR(-/0.585), SVM(-/0.593), RF(-/0.526), XGBoost(-/0.789),LightGBM(-/0.553),CatBoost(-/0.461) | - |
| Pérez-Tome JC et al^[76]^ | RF(-/0.969) | RF(-/0.98) | RF(-/0.97) | - | - | - | - | - |
| Rahman MS et al^[77]^ | MLP(-/0.98),XGBoost(-/0.98),RF(-/0.94),LR(-/0.99),adaBoost(-/0.90),GB(-/0.98),CatBoost(-/0.98),ET(-/0.99) | MLP(-/0.95),XGBoost(-/0.94),RF(-/0.94),LR(-/0.96),adaBoost(-/0.93),GB(-/0.93),CatBoost(-/0.93),ET(-/0.95) | - | MLP(-/0.94),XGBoost(-/0.94),RF(-/0.94),LR(-/0.94),adaBoost(-/0.94),GB(-/0.94),CatBoost(-/0.94),ET(-/0.95) | - | - | MLP(-/0.95),XGBoost(-/0.94),RF(-/0.94),LR(-/0.96),adaBoost(-/0.94),GB(-/0.93),CatBoost(-/0.94),ET(-/0.95) | - |
| Wang B et al^[78]^ | LR(0.820/0.849) | LR(0.846/0.842) | LR(0.897/-) | LR(0.599/-) | LR(0.529/0.468) | LR(0.917/0.947) | - | SOFA:0.753 |
| Selcuk M et al^[79]^ | MLP(-/0.8524), SVM(-/0.7635),LR(-/0.8236), RF(-/0.7707), XGB(-/0.7588), KNN(-/0.6172), DT(-/0.7514), GNB(-/0.6495) | - | MLP(-/0.7714), SVM(-/0.5714), LR(-/0.7285), RF(-/0.5857), XGB(-/0.6285), KNN(-/0.2714), DT(-/0.6285), GNB(-/0.3285) | MLP(-/0.9333), SVM(-/0.9555), LR(-/0.9185), RF(-/0.9555), XGB(-/0.8888), KNN(-/0.9629), DT(-/0.874), GNB(-/0.9703) | - | - | - | APACHE II:0.826;SAPS II:0.7887;SOFA:0.809 |
| Xie Y et al^[80]^ | LR(0.849/0.828) | - | - | - | - | - | - | - |
| Zheng YJ et al^[81]^ | LR(0.857/0.819) | - | - | - | - | - | - | APACHE II:0.756(0.717–0.792) |
| Wang S et al^[82]^ | TCF(0.955/0.733) | TCF(0.869/0.686) | TCF(0.905/0.640) | TCF(0.834/0.700) | - | - | TCF(0.874/0.458) | - |
| Chen T et al^[83]^ | LR(0.8241/0.7333),SVM(0.8541/0.8093),XGBoost(0.9699/0.9000),SWSELM(0.9760/0.9387) | LR(0.7914/0.7250), SVM(0.7976/0.7750),XGBoost(0.9018/0.8750),SWSELM(0.8957/0.9000) | LR(0.5593/0.7250),SVM(0.5085/0.7750),XGBoost(0.7797/1.0000),SWSELM(0.8136/0.8000) | LR(0.9231/0.8400),SVM(0.9615/0.8800),XGBoost(0.9712/0.8000),SWSELM(0.9423/0.9600) | - | - | LR(0.6600/0.5926),SVM(0.6452/0.6667),XGBoost(0.8519/0.8571),SWSELM(0.8496/0.8571) | - |
| Kurtkulagi O et al^[84]^ | LR(0.756/-) | - | - | - | - | - | - | - |
| Li Q et al^[85]^ | COX(-/0.790) | - | COX(-/0.816) | COX(-/0.344) | - | - | - | SOFA:0.582(0.516-0.649) |
| Shi S et al^[86]^ | LR(0.79/0.77), GNB(0.76/0.75), KNN(0.82/0.75), SVM(0.78/0.77), ANN(0.90/0.76),  Decision Tree(1.00/0.70), Random Forest(1.00/0.77), GBM(0.82/0.78), CatBoost(0.91/0.77) | - | - | - | - | - | - | - |
| Shi W et al^[87]^ | COX:4week(0.882/0.851),6week(0.836/0.820),8week(0.843/0.794) | - | - | - | - | - | - | - |
| Wang Y et al^[88]^ | XGBoost(-/0.873),LR(-/0.829),SVM(-/0.830),DNN(-/0.837) | XGBoost(-/0.777), LR(-/0.736), SVM(-/0.768), DNN(-/0.752) | XGBoost(-/0.818), LR(-/0.790), SVM(-/0.745), DNN(-/0.769) | XGBoost(-/0.768), LR(-/0.725), SVM(-/0.773), DNN(-/0.749) | XGBoost(-/0.818), LR(-/0.789), SVM(-/0.745), DNN(-/0.768) | XGBoost(-/0.768), LR(-/0.725), SVM(-/0.773), DNN(-/0.749) | XGBoost(-/0.551), LR(-/0.500), SVM(-/0.518), DNN(-/0.509) | SOFA:0.724(0.678-0.770);SAPII:0.706(0.659-0.753) |
| Yang Y et al^[89]^ | LR(-/0.849) | - | - | - | - | - | - | - |
| Zhang Y et al^[90]^ | LR(0.845/0.857) | - | LR(0.759/-) | LR(0.85/-) | - | - | - | - |
| Zhu XY et al^[91]^ | XGBoost(-/0.88),NN(-/0.86),SVM(-/0.85),NB(-/0.84),MLP(-/0.83),LR(-/0.82) | XGBoost(-/0.84), NN(-/0.83), SVM(-/0.82), NB(-/0.74), MLP(-/0.82), LR(-/0.82) | XGBoost(-/0.64), NN(-/0.62), SVM(-/0.62), NB(-/0.80), MLP(-/0.61), LR(-/0.59) | XGBoost(-/0.93), NN(-/0.93), SVM(-/0.91), NB(-/0.71), MLP(-/0.92), LR(-/0.92) | XGBoost(-/0.93), NN(-/0.93), SVM(-/0.91), NB(-/0.71), MLP(-/0.92), LR(-/0.92) | XGBoost(-/0.85), NN(-/0.84), SVM(-/0.84), NB(-/0.89), MLP(-/0.84), LR(-/0.83) | XGBoost(-/0.71), NN(-/0.70), SVM(-/0.69), NB(-/0.65), MLP(-/0.68), LR(-/0.66) | - |

Abbreviations: D, development; I, internal validation; APACHE II, acute physiology and chronic health evaluation II; SOFA, Sequential Organ Failure Assessment; SAPS, Simplified Acute Physiology Score; APS, Acute physiology score,; -, not reported.
