# Supplementary material for: Prediction models for mortality in patients with sepsis: a systematic review and meta-analysis
Source: Front Med (Lausanne). 2026 Jun 10;13:1730156. doi: 10.3389/fmed.2026.1730156 (PMC13290529; doi:10.3389/fmed.2026.1730156)
Supplement: Supplementary file 5 [file Table_5.DOC]

**Supplementary Table 3**

**The predictors for the included models**

| **No** | **Predictor factors** | **Fre.** | **No** | **Predictor factors** | **Fre.** | **No** | **Predictor factors** | **Fre.** |
| --- | --- | --- | --- | --- | --- | --- | --- | --- |
| 1 | Age | 19 | 45 | Magnesium | 2 | 89 | AIDS | 1 |
| 2 | Lactate | 13 | 46 | Peripheral vascular disease | 2 | 90 | Blood and not Urinary, Lung, or Abdominal Infection | 1 |
| 3 | Albumin | 8 | 47 | CRP | 2 | 91 | Aniongap_max | 1 |
| 4 | SOFA score | 8 | 48 | HCT | 2 | 92 | Bicarbonate | 1 |
| 5 | Vasopressor | 7 | 49 | Lung Infection | 2 | 93 | Paraplegia | 1 |
| 6 | Comorbidities with metastatic cancer | 6 | 50 | Chronic pulmonary disease | 2 | 94 | Pulmonary-circulatory | 1 |
| 7 | GCS score | 6 | 51 | Diabetes | 2 | 95 | Thrombocytopenia | 1 |
| 8 | TBIL | 5 | 52 | Obesity | 2 | 96 | Skin infection | 1 |
| 9 | PLT | 5 | 53 | Urinary tract Infection | 2 | 97 | CNS infection | 1 |
| 10 | Shock | 5 | 54 | Psychoses | 2 | 98 | Self-pay | 1 |
| 11 | RR | 4 | 55 | RDW | 2 | 99 | Black, non-Hispanic | 1 |
| 12 | SBP | 4 | 56 | D-Dimer | 2 | 100 | LOD(Logistic Organ Dys function score) | 1 |
| 13 | Chronic renal disease | 4 | 57 | NLR | 2 | 101 | 2nd episode | 1 |
| 14 | PCT | 4 | 58 | HR | 2 | 102 | 3–4 episodes | 1 |
| 15 | Creatinine | 4 | 59 | INR | 2 | 103 | Multiple sites | 1 |
| 16 | Mechanical ventilation | 4 | 60 | Anemia | 2 | 104 | SAPS II | 1 |
| 17 | BUN | 4 | 61 | Gram positive | 2 | 105 | McCabe | 1 |
| 18 | Calcium | 3 | 62 | Gram positive | 2 | 106 | 1 chronic illness | 1 |
| 19 | AST | 3 | 63 | Hemodialysis | 2 | 107 | ≥2 chronic illness | 1 |
| 20 | PaO2/FiO2 | 3 | 64 | Abdominal Infection | 2 | 108 | Dementia | 1 |
| 21 | Liver disease | 3 | 65 | PH | 2 | 109 | L/A | 1 |
| 22 | APACHE II score | 3 | 67 | Potassium | 2 | 110 | IL-6 | 1 |
| 23 | Na | 3 | 66 | Phosphate | 1 | 111 | Neutrophils | 1 |
| 24 | PT | 3 | 68 | cerebrovascular disease | 1 | 112 | Lymphocytes | 1 |
| 25 | Respiratory failure | 3 | 69 | glucocorticoids | 1 | 113 | Monocytes | 1 |
| 26 | temperature | 3 | 70 | PLS index | 1 | 114 | OASIS | 1 |
| 27 | SPO2 | 3 | 71 | FBG | 1 | 115 | NGAL | 1 |
| 28 | Lymphoma | 3 | 72 | Calprotectin clearance | 1 | 116 | Fetuin-A | 1 |
| 29 | BNP | 3 | 73 | Ethnicity | 1 | 117 | RBCV | 1 |
| 30 | Gender(female) | 2 | 74 | Hemoglobin | 1 | 118 | IL-6D3 | 1 |
| 31 | LDH | 2 | 75 | ALP | 1 | 119 | PCTD1 | 1 |
| 32 | Serum phosphorus | 2 | 76 | Elixhauser score | 1 | 120 | LACcD3 | 1 |
| 33 | Hypertension | 2 | 77 | BE | 1 | 121 | Central venous catheterization | 1 |
| 34 | Congestive heart failure | 2 | 78 | PLR | 1 | 122 | CCI | 1 |
| 35 | Neurological disorders | 2 | 79 | LMR | 1 | 123 | APTT | 1 |
| 36 | Weight loss | 2 | 80 | WBC | 1 | 124 | NEWS | 1 |
| 37 | Hypothyroidism | 2 | 81 | Race | 1 | 125 | PIRO | 1 |
| 38 | suPAR | 2 | 82 | Admission type of internal medicine | 1 | 126 | Intubation | 1 |
| 39 | Cholinesterase | 2 | 83 | Renal replacement therapy | 1 | 127 | Antiarrhythmic drugs | 1 |
| 40 | MAP | 2 | 84 | TM | 1 | 128 | Digestive tract infection | 1 |
| 41 | Urine output | 2 | 85 | Solid tumor without metastasis | 1 | 129 | Other infections | 1 |
| 42 | Depression | 2 | 86 | Alcohol abuse | 1 | 130 | MV days | 1 |
| 43 | Drug abuse | 2 | 87 | Paralysis | 1 | 131 | MV | 1 |
| 44 | CU care | 2 | 88 | Peptic ulcer disease | 1 |  |  |  |
