# Supplementary material for: Prediction models for mortality in patients with sepsis: a systematic review and meta-analysis
Source: Front Med (Lausanne). 2026 Jun 10;13:1730156. doi: 10.3389/fmed.2026.1730156 (PMC13290529; doi:10.3389/fmed.2026.1730156)
Supplement: Supplementary file 6 [file Table_6.DOC]

**Table 3** **Details of the external validation studies of prediction models**

| **Author** | **Dataset source** | **Type of external validation** | **Sample size(cases/total)** | **Study period** | **Age**  **(years)** | **Gender (Male/Female)** | **AUC(95% CI)** |
| --- | --- | --- | --- | --- | --- | --- | --- |
| Zhi D et al^[8]^ | Beijing Friendship Hospital, Capital Medical University | Completely independent datasets | 59/125 | 2018.3-2020.3 | 65.50 ± 14.52 | 90/35 | 0.618(0.519-0.717) |
| Li F et al^[13]^ | ali University’s First Affiliated Hospital over the same period | Institutional/geographical split | 37/75 | 2018.1.1-2022.12.31 | 61（44-71） | 50/25 | 0.91(0.82-0.96) |
| Zhang K et al^[20]^ | eICU | Completely independent datasets | 1,042/5,658 | 2014-2015 | 67(56-78) | 3011/2647 | 0.775 |
| Zeng Z et al^[21]^ | MIMIC-III | Completely independent datasets | 2,169/12,905 | 2001-2012 | 64.4±16.3 | 6988/5917 | 0.815(0.808-0.822) |
| Lagu T et al^[33]^ | Project IMPACT database | Completely independent datasets | 116/357 | 2002-2009 | 65（52-77） | 203/154 | 0.693 |
| Ford DW et al^[40]^ | Martin approach; Angus approach | Completely independent datasets | 78,309/662,739 | 2012 | / | / | 0.803 |
| Gong M et al^[43]^ | EICU | Completely independent datasets | 2,597/29,620 | 2015-2015 | 64.64±14.95 | 17844/11776 | 0.78 |
| Zhuang J et al^[49]^ | MIMIC-IV, eICU, Zigong | Completely independent datasets | 1,664/15,532 (MIMIC-IV); 2,494/22,617 (eICU);423/1,198(Zigong) | 2008-2019 (MIMIC-IV),2014-2015 (eICU), 2019-2020 (Zigong) | / | / | 0.87(MIMIC-IV),0.83(eICU),0.68(Zigong) |
| Zheng F et al^[50]^ | eICU | Completely independent datasets | 1,224/6,649 | 2014-2015 | / | / | 0.7603(0.7413-0.7794) |
| Bao C et al^[53]^ | eICU-CRD | Completely independent datasets | 1,491/8,819 | 2014-2015 | 67(56-78) | 4532/4287 | 0.85(0.74-0.96) |
| Wang ZY et al^[54]^ | MIMIC-III | Completely independent datasets | 889/4,560 | 2001-2012 | / | / | 0.883 |
| Chicco D et al^[59]^ | South Korean critically ill patients | Completely independent datasets | 24/137 | 2007-2015 | / | 90/47 | 0.899 |
| Cheng CY et al^[62]^ | Chang Gung Medical Center database (5 EDs, Taiwan) | Institutional/geographical split | 3,860/38,658 | 2017 | / | / | 0.811(0.766-0.857) |
| Pérez-Tome JC et al^[76]^ | MIMIC III | Completely independent datasets | 741/4,559 | 2001-2012 | / | / | 0.973(0.968-0.981) |
| Wang S et al^[82]^ | Cohort 1-1 Pediatric ICU; Cohort 1-2 Respiratory ICU; Cohort 2; Cohort 3 | Institutional/geographical split | 287/1,421 | 2003.2-2023.11 | / | / | 0.786(0.725-0.84) |
| Shi S et al^[86]^ | ICU, Fujian Provincial Hospital, China | Completely independent datasets | 94/325 | 2023.1-2023.11 | / | / | 0.77 |
| Wang Y et al^[88]^ | First Affiliated Hospital of Wenzhou Medical University ICU | Completely independent datasets | 81/487 | 2021.1-2022.6 | / | / | 0.844(0.81-0.878) |
| Yang Y et al^[89]^ | ICU of tertiary hospital in Xinjiang | Temporal split | 115/242 | 2023.1-2024.6 | 61.2±15.7 | 144/98 | 0.837(0.787-0.886) |

Note: Type of external validation was classified as temporal split, institutional/geographical split, or completely independent datasets based on the relationship between the development cohort and the reported external validation cohort(s) in the original studies.
