# Supplementary material for: Prediction models for mortality in patients with sepsis: a systematic review and meta-analysis
Source: Front Med (Lausanne). 2026 Jun 10;13:1730156. doi: 10.3389/fmed.2026.1730156 (PMC13290529; doi:10.3389/fmed.2026.1730156)
Supplement: Supplementary file 7 [file Table_7.DOC]

**Supplementary Table 4**

**Supplementary Table 4.1 Risk of bias assessment (****PROBAST+**AI) model development.

| Author | year | Domain1(Participants and data sources) | | Domain 2 (Predictors) | | Domain 3 (Outcome) | | Domain 4(Analysis)^g^ | Overall judgement | |
| --- | --- | --- | --- | --- | --- | --- | --- | --- | --- | --- |
|  |  | Risk of Bias^a^ | Applicability^b^ | Risk of Bias^c^ | Applicability^d^ | Risk of Bias^e^ | Applicability^f^ |  | Risk of Bias^h^ | Applicability^i^ |
| Zhi D et al | 2021 | low | low | low | low | low | low | high | high | low |
| Zhang G et al | 2024 | low | low | low | low | low | low | low | low | low |
| Yu Z et al | 2024 | low | low | high | low | low | low | high | high | low |
| Li Y et al | 2024 | low | low | high | low | low | low | high | high | low |
| Xu Z et al | 2024 | low | low | high | low | low | low | high | high | low |
| Li F et al | 2024 | low | low | low | low | low | low | high | high | low |
| García de Guadiana-Romualdo L et al | 2024 | low | low | low | low | low | low | high | high | low |
| Li F et al | 2023 | low | low | low | low | low | low | low | low | low |
| Wang J et al | 2023 | low | low | high | low | low | low | high | high | low |
| Taylor RA et al | 2016 | low | low | low | low | low | low | high | high | low |
| Seo MH et al | 2016 | low | low | high | low | low | low | high | high | low |
| Zhao C et al | 2019 | low | low | high | low | low | low | high | high | low |
| Zhang K et al | 2021 | low | low | low | low | low | low | low | low | low |
| Zeng Z et al | 2021 | low | low | high | low | low | low | low | high | low |
| Zeng Q et al | 2021 | low | low | high | low | low | low | high | high | low |
| Wernly B et al | 2020 | low | low | low | low | low | low | low | low | low |
| Wang H et al | 2022 | low | low | low | low | low | low | high | high | low |
| van Doorn WPTM et al | 2021 | low | low | low | low | low | low | low | low | low |
| Su L et al | 2021 | low | low | low | low | low | low | high | high | low |
| Rodríguez A et al | 2021 | low | low | low | low | low | low | high | high | low |
| Perng JW et al | 2019 | low | low | low | low | low | low | high | high | low |
| Park JY et al | 2022 | high | low | low | low | low | low | low | high | low |
| Liu N et al | 2021 | low | low | high | low | low | low | high | high | low |
| Liu H et al | 2021 | low | low | high | low | low | low | high | low | low |
| Li K et al | 2021 | low | low | low | low | low | low | high | high | low |
| Lagu T et al | 2011 | low | low | low | low | low | low | low | low | low |
| Kong G et al | 2020 | low | low | low | low | low | low | high | high | low |
| Karlsson A et al | 2021 | low | low | high | low | low | low | high | high | low |
| Hu C et al | 2022 | low | low | low | low | low | low | high | high | low |
| Hou N et al | 2020 | low | low | high | low | low | low | high | high | low |
| Hargovan S et al | 2021 | low | low | high | low | low | low | high | high | low |
| García-Gallo JE et al | 2020 | low | low | low | low | low | low | high | high | low |
| Ford DW et al | 2016 | low | low | low | low | low | low | low | low | low |
| Phillips GS et al | 2018 | low | low | high | low | low | low | high | high | low |
| Ribas Ripoll VJ et al | 2014 | low | low | low | low | low | low | high | high | low |
| Gong M et al | 2022 | low | low | low | low | low | low | low | low | low |
| García-Gallo J E et al | 2019 | low | low | low | low | low | low | high | high | low |
| Wang W et al | 2021 | low | low | high | low | low | low | high | high | low |
| Ding X et al | 2022 | low | low | high | low | low | low | high | high | low |
| Wang L et al | 2022 | low | low | low | low | low | low | high | high | low |
| Cheng YW et al | 2024 | low | low | low | low | low | low | low | low | low |
| Zhuang J et al | 2023 | low | low | low | low | low | low | low | low | low |
| Zheng F et al | 2023 | low | low | low | low | low | low | low | low | low |
| Pan X et al | 2023 | low | low | high | low | low | low | high | high | low |
| Li S et al | 2023 | low | low | low | low | low | low | low | low | low |
| Bao C et al | 2022 | low | low | low | low | low | low | low | low | low |
| Wang ZY et al | 2022 | low | low | high | low | low | low | high | high | low |
| Su Y et al | 2022 | low | low | high | low | low | low | high | high | low |
| Ke X et al | 2022 | low | low | low | low | low | low | low | low | low |
| Zhang Z et al | 2017 | high | low | low | low | low | low | high | high | low |
| Wang M et al | 2023 | low | low | high | low | low | low | high | high | low |
| Chicco D et al | 2020 | low | high | low | high | low | high | low | low | high |
| Adrie C et al | 2009 | low | low | low | low | low | low | high | high | low |
| Chen SH et al | 2021 | low | low | high | low | low | low | high | high | low |
| Cheng CY et al | 2022 | low | low | low | low | low | low | high | high | low |
| Gao J et al | 2024 | low | low | low | low | low | low | high | high | low |
| Greco, M et al | 2023 | low | low | low | low | low | low | high | high | low |
| He B et al | 2024 | low | low | low | low | low | low | high | high | low |
| Hong C et al | 2024 | low | low | low | low | low | low | high | high | low |
| Jeon E et al | 2023 | low | low | high | low | low | low | low | high | low |
| Jiang Z et al | 2023 | low | low | low | low | low | low | high | high | low |
| Koozi H et al | 2023 | low | low | high | low | low | low | high | high | low |
| Li M et al | 2022 | low | low | high | low | low | low | high | high | low |
| Li Y et al | 2023 | low | low | high | low | low | low | low | high | low |
| Lin XM et al | 2024 | low | low | low | low | low | low | high | high | low |
| Liu Y et al | 2022 | low | low | high | low | low | low | high | high | low |
| Lu B et al | 2023 | low | low | high | low | low | low | high | high | low |
| Park SW et al | 2024 | low | low | low | low | low | low | low | low | low |
| Pérez-Tome JC et al | 2024 | low | low | low | low | low | low | high | high | low |
| Rahman MS et al | 2024 | low | low | high | low | low | low | high | high | low |
| Wang B et al | 2024 | low | low | high | low | low | low | high | high | low |
| Selcuk M et al | 2022 | low | low | low | low | low | low | high | high | low |
| Xie Y et al | 2023 | low | low | high | low | low | low | high | high | low |
| Zheng YJ et al | 2022 | low | low | high | low | low | low | high | high | low |
| Wang S et al | 2025 | low | low | high | low | low | low | low | low | low |
| Chen T et al | 2025 | low | low | high | low | low | low | high | high | low |
| Kurtkulagi O et al | 2025 | low | low | low | low | low | low | high | high | low |
| Li Q et al | 2025 | low | low | high | low | low | low | high | high | low |
| Shi S et al | 2025 | low | low | low | low | low | low | low | low | low |
| Shi W et al | 2025 | low | low | low | low | low | low | high | high | low |
| Wang Y et al | 2025 | low | low | low | low | low | low | low | low | low |
| Yang Y et al | 2025 | low | low | high | low | low | low | low | high | low |
| Zhang Y et al | 2025 | low | low | high | low | low | low | low | high | low |
| Zhu XY et al | 2025 | low | low | high | low | low | low | low | high | low |

**Abbreviation:** PROBAST+AI, prediction model risk of bias assessment tool for artificial intelligence, L low; H high; U unclear.

**Footnote:** Signaling questions are rated as "yes" (Y), "probably yes" (PY), "probably no" (PN), "no" (N), "no information" (NI), and in some cases "not applicable" (NA). All signaling questions are phrased in such a way that "yes" or "probably yes" indicates a low risk of bias. Any signaling questions rated as "no" or "probably no" indicate a potential high risk of bias in that domain. If there are no "no" or "probably no" ratings, but "no information" (NI) is present, the risk of bias in that domain is classified as unclear.

**a. Participants and data sources** **(Risk of Bias)**

1.1 Was an appropriate study design used?

1.2 Did the in- and exclusions of study participants result in a representative dataset?

**e. Participants and data sources** **(Applicability)**

Concern that the (data of the) included participants do not match the review question or the assessor’s intended use of the prediction model.

**b. Predictors (Risk of Bias)**

2.1 Were predictors defined and assessed in a similar way for all participants?

2.2 Was any pre-processing of predictors similar for all participants?

**f. Predictors (Applicability)**

Concern that the definition, pre-processing, assessment, or timing of assessment of the predictors in the model do not match the review question or the assessor’s intended use.

**c. Outcome** **(Risk of Bias)**

3.1 Were outcomes defined and assessed appropriately?

3.2 Were outcomes defined and assessed in a similar way for all participants?

3.3 Were outcome assessments made without use or knowledge of predictor data?

**g. Outcome (Applicability)**

Concern that the outcome, its definition, assessment, or timing of assessment do not match the review question or the assessor’s intended use.

**d. Analysis (Risk of Bias)**

4.1 Was the sample size greater than 50?

4.2 Were continuous and categorical predictors handled appropriately?

4.3 Were methods used to address potential model overfitting?

**h.** **Overall judgement (Risk of Bias)**

Low risk: If all four domains were rated low concern regarding quality.

High risk: If at least one domain was rated high concern regarding quality.

Unclear: If at least one domain was rated unclear concern regarding quality and no domains were rated high concern.

**i.** **Overall judgement (Applicability)**

Low risk: If all three domains were rated low concern for applicability.

High risk: If at least one domain was rated high concern for applicability.

Unclear: If at least one domain was rated unclear concern for applicability and no domains were rated high concern.

**Supplementary Table 4.2 Risk of bias assessment (PROBAST+**AI) model evaluation.

| Author | year | Domain1(Participants and data sources) | | Domain 2 (Predictors) | | Domain 3 (Outcome) | | Domain 4(Analysis)^g^ | Overall judgement | |
| --- | --- | --- | --- | --- | --- | --- | --- | --- | --- | --- |
|  |  | Risk of Bias^a^ | Applicability^b^ | Risk of Bias^c^ | Applicability^d^ | Risk of Bias^e^ | Applicability^f^ |  | Risk of Bias^h^ | Applicability^i^ |
| Zhi D et al | 2021 | low | low | low | low | low | low | high | high | low |
| Zhang G et al | 2024 | low | low | low | low | low | low | low | low | low |
| Yu Z et al | 2024 | low | low | low | low | low | low | high | high | low |
| Li Y et al | 2024 | low | low | low | low | low | low | high | high | low |
| Xu Z et al | 2024 | low | low | low | low | low | low | high | high | low |
| Li F et al | 2023 | low | low | low | low | low | low | low | low | low |
| Wang J et al | 2023 | low | low | low | low | low | low | high | high | low |
| Taylor RA et al | 2016 | low | low | low | low | low | low | high | high | low |
| Seo MH et al | 2016 | low | low | low | low | low | low | high | high | low |
| Zhao C et al | 2019 | low | low | low | low | low | low | high | high | low |
| Zhang K et al | 2021 | low | low | low | low | low | low | low | low | low |
| Zeng Z et al | 2021 | low | low | low | low | low | low | low | low | low |
| Zeng Q et al | 2021 | low | low | low | low | low | low | high | high | low |
| Wernly B et al | 2020 | low | low | low | low | low | low | low | low | low |
| Wang H et al | 2022 | low | low | low | low | low | low | high | high | low |
| van Doorn WPTM et al | 2021 | low | low | low | low | low | low | low | low | low |
| Su L et al | 2021 | low | low | low | low | low | low | high | high | low |
| Rodríguez A et al | 2021 | low | low | low | low | low | low | high | high | low |
| Perng JW et al | 2019 | low | low | low | low | low | low | high | high | low |
| Park JY et al | 2022 | high | low | low | low | low | low | low | high | low |
| Liu N et al | 2021 | low | low | low | low | low | low | high | high | low |
| Liu H et al | 2021 | low | low | low | low | low | low | high | high | low |
| Li K et al | 2021 | low | low | low | low | low | low | high | high | low |
| Lagu T et al | 2011 | low | low | low | low | low | low | low | low | low |
| Kong G et al | 2020 | low | low | low | low | low | low | high | high | low |
| Karlsson A et al | 2021 | low | low | low | low | low | low | high | high | low |
| Hu C et al | 2022 | low | low | low | low | low | low | high | high | low |
| Hou N et al | 2020 | low | low | low | low | low | low | high | high | low |
| Hargovan S et al | 2021 | low | low | low | low | low | low | low | low | low |
| García-Gallo JE et al | 2020 | low | low | low | low | low | low | high | high | low |
| Ford DW et al | 2016 | low | low | low | low | low | low | low | low | low |
| Phillips GS et al | 2018 | low | low | low | low | low | low | high | high | low |
| Ribas Ripoll VJ et al | 2014 | low | low | low | low | low | low | high | high | low |
| Gong M et al | 2022 | low | low | low | low | low | low | low | low | low |
| García-Gallo J E et al | 2019 | low | low | low | low | low | low | high | high | low |
| Cheng YW et al | 2024 | low | low | low | low | low | low | low | low | low |
| Zhuang J et al | 2023 | low | low | low | low | low | low | low | low | low |
| Zheng F et al | 2023 | low | low | low | low | low | low | low | low | low |
| Pan X et al | 2023 | low | low | low | low | low | low | high | high | low |
| Li S et al | 2023 | low | low | low | low | low | low | low | low | low |
| Bao C et al | 2022 | low | low | low | low | low | low | low | low | low |
| Wang ZY et al | 2022 | low | low | low | low | low | low | high | high | low |
| Su Y et al | 2022 | low | low | low | low | low | low | high | high | low |
| Ke X et al | 2022 | low | low | low | low | low | low | low | low | low |
| Zhang Z et al | 2017 | high | low | low | low | low | low | high | high | low |
| Wang M et al | 2023 | low | low | low | low | low | low | high | high | low |
| Chicco D et al | 2020 | low | high | low | high | low | high | low | low | high |
| Adrie C et al | 2009 | low | low | low | low | low | low | high | high | low |
| Cheng CY et al | 2022 | low | low | low | low | low | low | high | high | low |
| Gao J et al | 2024 | low | low | low | low | low | low | high | high | low |
| Greco, M et al | 2023 | low | low | low | low | low | low | high | high | low |
| He B et al | 2024 | low | low | low | low | low | low | high | high | low |
| Hong C et al | 2024 | low | low | low | low | low | low | high | high | low |
| Jeon E et al | 2023 | low | low | low | low | low | low | low | low | low |
| Jiang Z et al | 2023 | low | low | low | low | low | low | high | high | low |
| Koozi H et al | 2023 | low | low | low | low | low | low | high | high | low |
| Li Y et al | 2023 | low | low | low | low | low | low | low | low | low |
| Lin XM et al | 2024 | low | low | low | low | low | low | high | high | low |
| Liu Y et al | 2022 | low | low | low | low | low | low | high | high | low |
| Lu B et al | 2023 | low | low | low | low | low | low | high | high | low |
| Park SW et al | 2024 | low | low | low | low | low | low | low | low | low |
| Pérez-Tome JC et al | 2024 | low | low | low | low | low | low | high | high | low |
| Rahman MS et al | 2024 | low | low | low | low | low | low | high | high | low |
| Wang B et al | 2024 | low | low | low | low | low | low | high | high | low |
| Selcuk M et al | 2022 | low | low | low | low | low | low | high | high | low |
| Xie Y et al | 2023 | low | low | low | low | low | low | high | high | low |
| Zheng YJ et al | 2022 | low | low | low | low | low | low | high | high | low |
| Wang S et al | 2025 | low | low | low | low | low | low | low | low | low |
| Chen T et al | 2025 | low | low | low | low | low | low | high | high | low |
| Li Q et al | 2025 | low | low | low | low | low | low | high | high | low |
| Shi S et al | 2025 | low | low | low | low | low | low | low | low | low |
| Shi W et al | 2025 | low | low | low | low | low | low | high | high | low |
| Wang Y et al | 2025 | low | low | low | low | low | low | low | low | low |
| Yang Y et al | 2025 | low | low | low | low | low | low | low | low | low |
| Zhang Y et al | 2025 | low | low | low | low | low | low | low | low | low |
| Zhu XY et al | 2025 | low | low | low | low | low | low | low | low | low |

**Abbreviation:** PROBAST+AI, prediction model risk of bias assessment tool for artificial intelligence, L low; H high; U unclear.

Footnote: Signaling questions are rated as "yes" (Y), "probably yes" (PY), "probably no" (PN), "no" (N), "no information" (NI), and in some cases "not applicable" (NA). All signaling questions are phrased in such a way that "yes" or "probably yes" indicates a low risk of bias. Any signaling questions rated as "no" or "probably no" indicate a potential high risk of bias in that domain. If there are no "no" or "probably no" ratings, but "no information" (NI) is present, the risk of bias in that domain is classified as unclear.

**a. Participants and data sources (Risk of Bias)**

- 1. Was an appropriate study design used?
  2. Did the in- and exclusions of study participants result in a representative dataset?

**e. Participants and data sources(Applicability)**

Concern that the (data of the) included participants do not match the review question or the assessor’s intended use of the prediction model.

**b. Predictors (Risk of Bias)**

2.1 Were predictors defined and assessed in a similar way for all participants?

2.2 Was any pre-processing of predictors similar for all participants?

**f. Predictors(Applicability)**

Concern that the definition, pre-processing, assessment, or timing of assessment of the predictors in the model do not match the review question or the assessor’s intended use.

**c. Outcome (Risk of Bias)**

3.1 Were outcomes defined and assessed appropriately?

3.2 Were outcomes defined and assessed in a similar way for all participants?

3.3 Were outcome assessments made without use or knowledge of predictor data?

**g. Outcome(Applicability)**

Concern that the outcome, its definition, assessment, or timing of assessment do not match the review question or the assessor’s intended use.

**d. Analysis (Risk of Bias)**

4.1 Was model evaluation based on only apparent performance avoided?

4.2 Was the sample size greater than 50?

4.3 If data splitting was done to create training and test datasets, was there evidence that data leakage was avoided?

4.4 If resampling methods were used to evaluate model performance, were all model development steps replicated in the resampling process?

4.5 Was the predictive performance of the model evaluated appropriately, e.g., receiver operating characteristic curve?

**h. Overall judgement (Risk of Bias)**

Low risk: If all four domains were rated low risk of bias.

High risk: If at least one domain was rated high risk of bias.

Unclear: If at least one domain was rated unclear risk of bias and no domains were rated high risk of bias.

**i. Overall judgement (Applicability)**

Low risk: If all three domains were rated low concern for applicability.

High risk: If at least one domain was rated high concern for applicability.

Unclear: If at least one domain was rated unclear concern for applicability and no domains were rated high concern.
