# Supplementary material for: Prediction models for mortality in patients with sepsis: a systematic review and meta-analysis
Source: Front Med (Lausanne). 2026 Jun 10;13:1730156. doi: 10.3389/fmed.2026.1730156 (PMC13290529; doi:10.3389/fmed.2026.1730156)
Supplement: Supplementary file 8 [file Table_8.DOCX]

**Supplementary Table 5**

**Sensitivity analysis of mortality prediction models in external validation studies**

| Deletion | Result |
| --- | --- |
| Zhi D et al (2021) | AUC =0.804, 95% CI [0.764, 0.845] |
| Li F et al (2023) | AUC =0.785, 95% CI [0.745, 0.826] |
| Lagu T et al (2011) | AUC =0.803, 95% CI [0.763, 0.845] |
| Ford DW et al (2016) | AUC =0.794, 95% CI [0.755, 0.834] |
| Gong M et al (2022) | AUC =0.794, 95% CI [0.746, 0.844] |
| Zhuang J et al (2023) | AUC =0.786, 95% CI [0.749, 0.825] |
| Zhuang J et al (2023) | AUC =0.807, 95% CI [0.770, 0.847] |
| Chicco D et al (2020) | AUC =0.786, 95% CI [0.746, 0.828] |
| Cheng CY et al (2022) | AUC =0.792, 95% CI [0.751, 0.834] |
| Wang S et al (2025) | AUC =0.794, 95% CI [0.753, 0.837] |
| Shi S et al (2025) | AUC =0.796, 95% CI [0.755, 0.839] |
| Wang Y et al (2025) | AUC =0.789, 95% CI [0.747, 0.833] |
| Yang Y et al (2025) | AUC =0.789, 95% CI [0.748, 0.836] |
