# Supplementary material for: Prediction models for mortality in patients with sepsis: a systematic review and meta-analysis
Source: Front Med (Lausanne). 2026 Jun 10;13:1730156. doi: 10.3389/fmed.2026.1730156 (PMC13290529; doi:10.3389/fmed.2026.1730156)
Supplement: Supplementary file 9 [file Table_9.DOCX]

**Supplementary Table 6**

**The meta-analysis results of sepsis mortality in the included studies**

| **Groups** | **Model** | **Pooled estimate** | **Heterogeneity** | |
| --- | --- | --- | --- | --- |
|  |  | **(95%CI)** | ***I*^2^** | ***P*** |
| 28-days | Random | 0.302(0.267-0.337) | 98.5% | 0.000 |
| 30-days | Random | 0.211(0.157-0.265) | 98.7% | 0.000 |
| 90-days | Random | 0.436(0.194-0.677) | 94.2% | 0.000 |
| 1-year | Random | 0.497(0.372-0.622) | 99.0% | 0.000 |
| in-hospital | Random | 0.202(0.178-0.255) | 99.9% | 0.000 |
