# Supplementary material for: Prediction models for mortality in patients with sepsis: a systematic review and meta-analysis
Source: Front Med (Lausanne). 2026 Jun 10;13:1730156. doi: 10.3389/fmed.2026.1730156 (PMC13290529; doi:10.3389/fmed.2026.1730156)
Supplement: Supplementary file 11 [file Image_1.pdf]

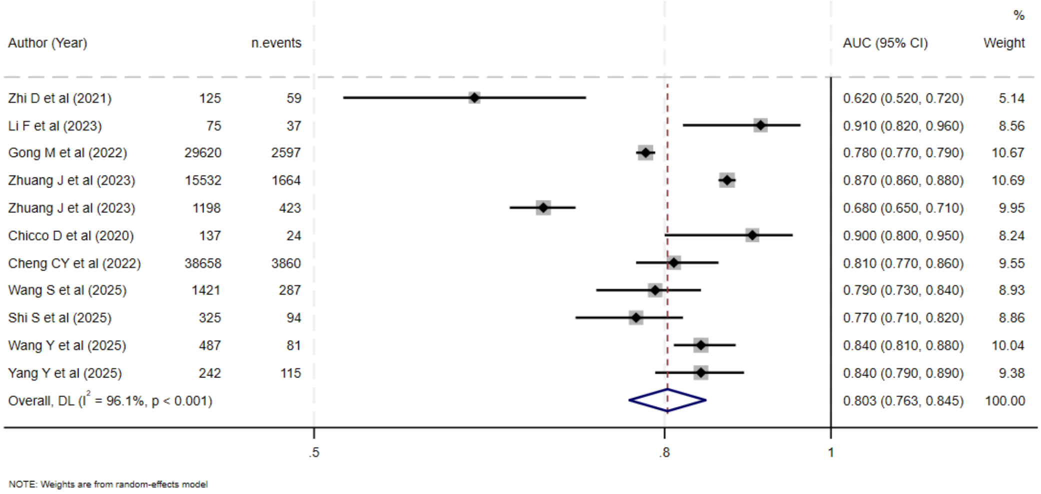

Supplementary Figure 1 The meta-analysis results of externally validated sepsis mortality prediction models using the Sepsis-3 criteria
