# Supplementary material for: Prediction models for mortality in patients with sepsis: a systematic review and meta-analysis
Source: Front Med (Lausanne). 2026 Jun 10;13:1730156. doi: 10.3389/fmed.2026.1730156 (PMC13290529; doi:10.3389/fmed.2026.1730156)
Supplement: Supplementary file 13 [file Image_3.pdf]

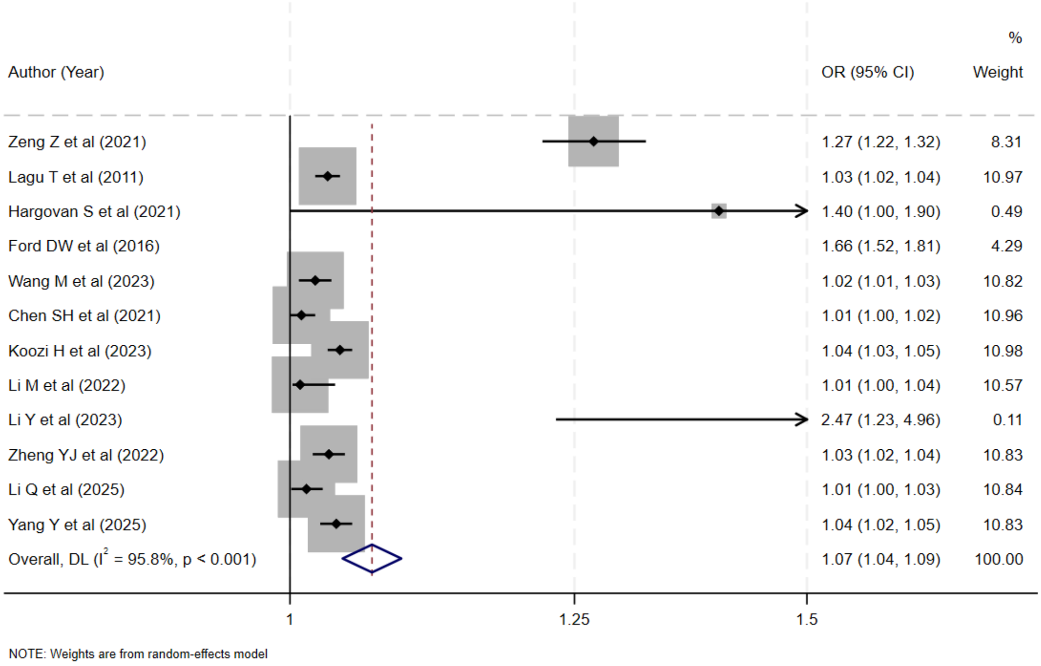

The meta-analysis results for age in the included studies

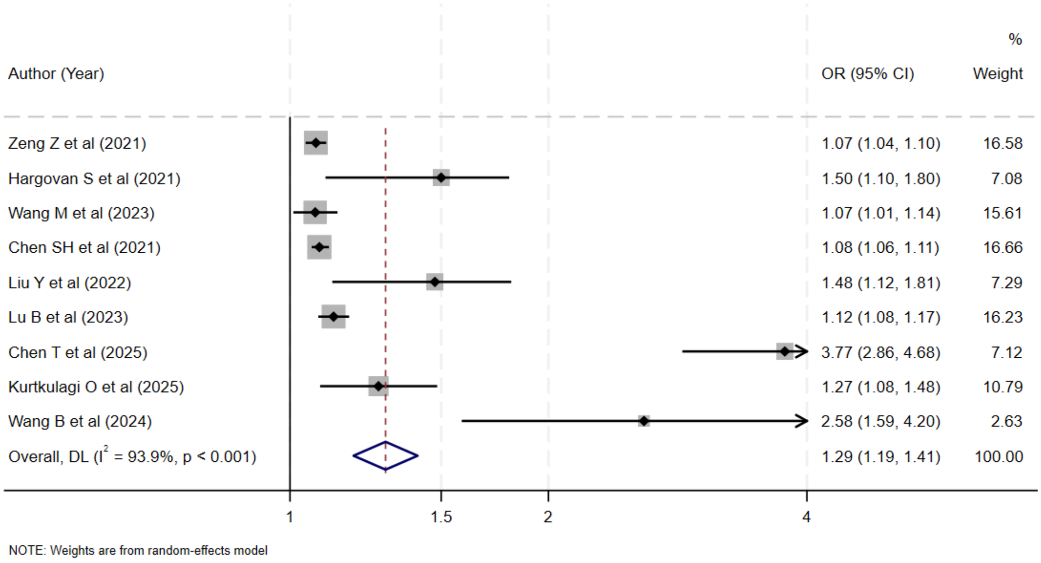

The meta-analysis results for lactate in the included studies

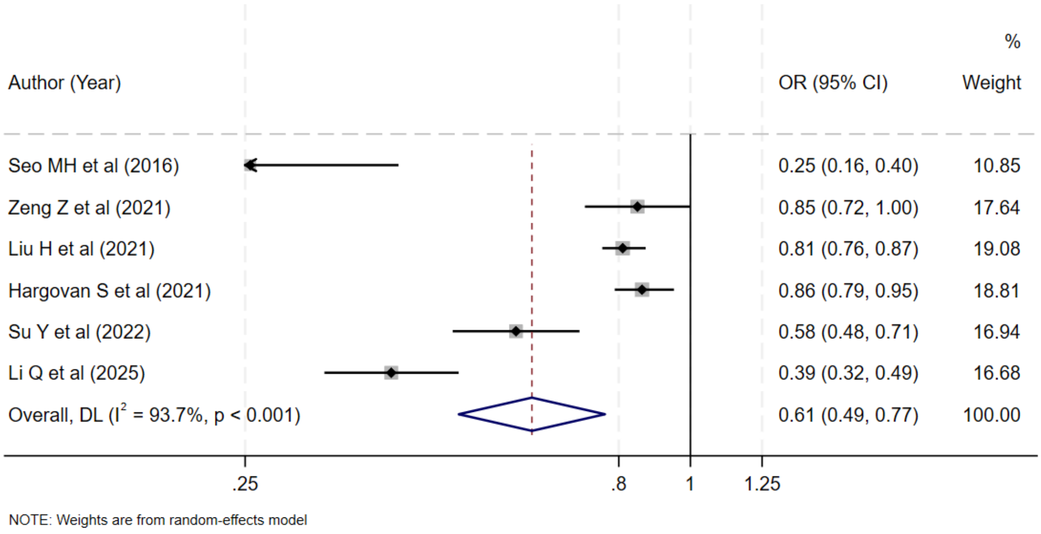

The meta-analysis results for albumin in the included studies

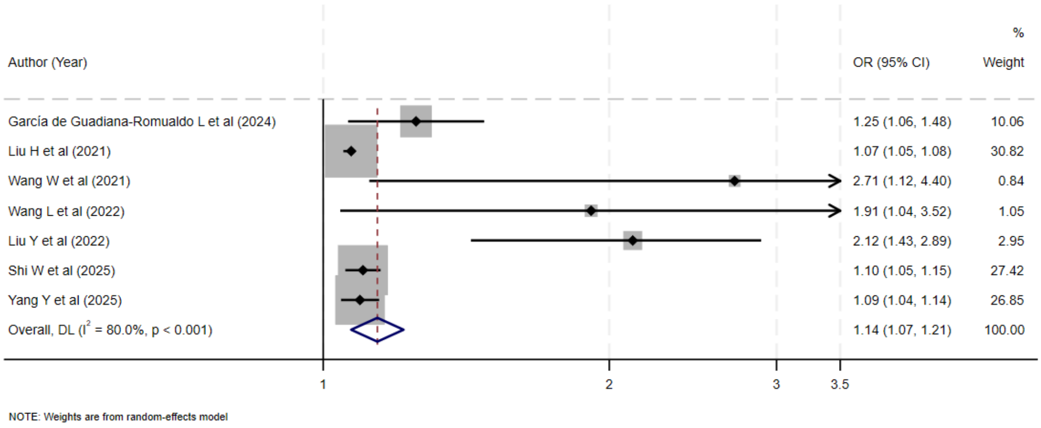

The meta-analysis results for SOFA scores in the included studies

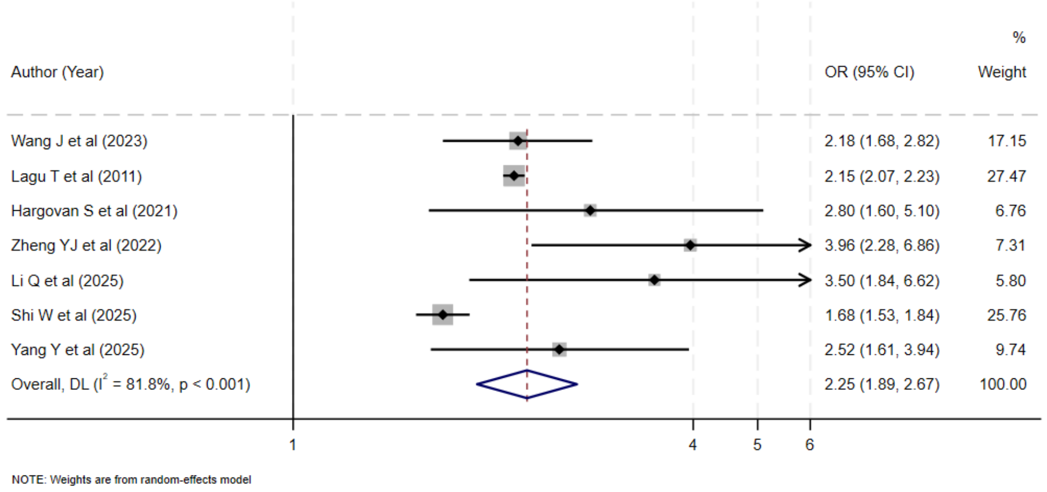

The meta-analysis results for vasopressor use in the included studies

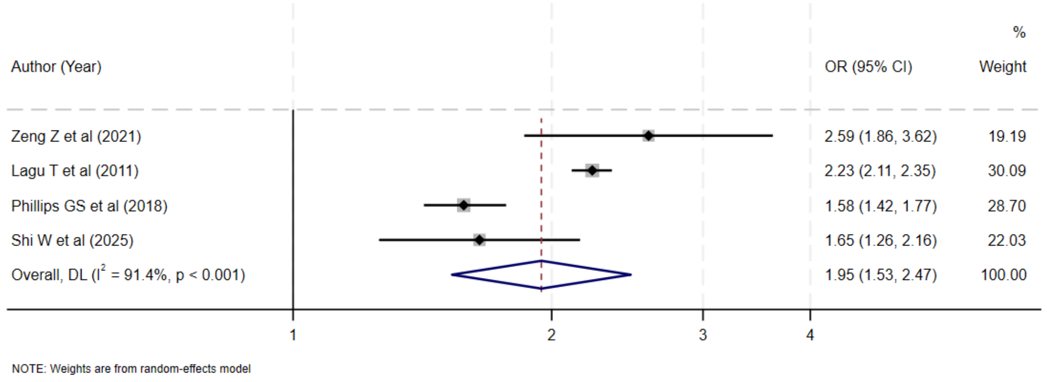

The meta-analysis results for comorbidities with metastatic cancer in the included studies

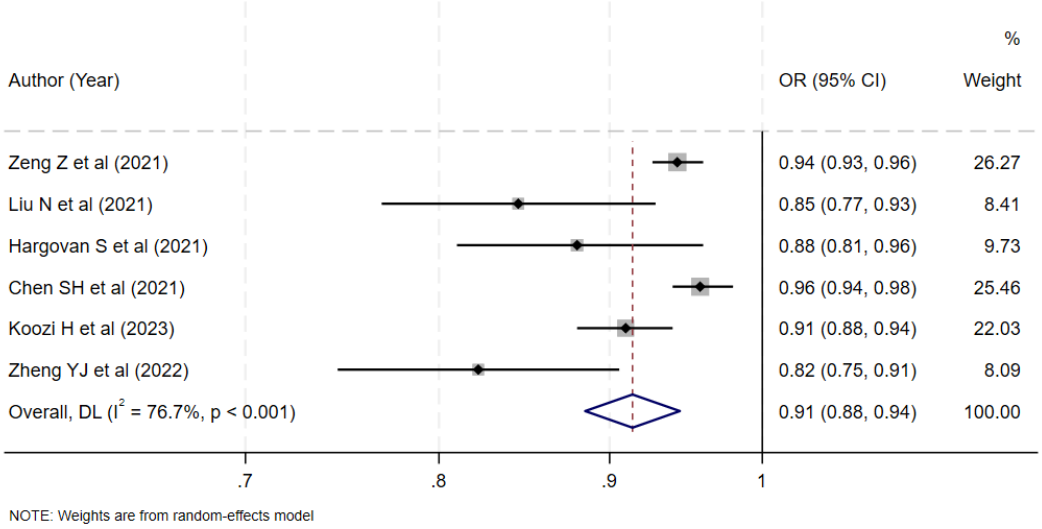

The meta-analysis results for GCS scores in the included studies
